# Elucidating acquired PARP inhibitor resistance in advanced prostate cancer

**DOI:** 10.1016/j.ccell.2024.10.015

**Published:** 2024-11-21

**Authors:** George Seed, Nick Beije, Wei Yuan, Claudia Bertan, Jane Goodall, Arian Lundberg, Matthew Tyler, Ines Figueiredo, Rita Pereira, Chloe Baker, Denisa Bogdan, Lewis Gallagher, Jan-Phillipp Cieslik, Semini Greening, Maryou Lambros, Rui Neves, Lorena Magraner-Pardo, Gemma Fowler, Berni Ebbs, Susana Miranda, Penny Flohr, Diletta Bianchini, Pasquale Rescigno, Nuria Porta, Emma Hall, Bora Gurel, Nina Tunariu, Adam Sharp, Stephen Pettit, Nikolas H. Stoecklein, Shahneen Sandhu, David Quigley, Christopher J. Lord, Joaquin Mateo, Suzanne Carreira, Johann de Bono

**Affiliations:** 1 The Institute of Cancer Research, London, UK; 2 The Royal Marsden NHS Foundation Trust, London, UK; 3 Heinrich Heine University, Duüsseldorf, Germany; 4 UCSF, San Francisco, CA, USA; 5 Vall d’Hebron Institute of Oncology, Barcelona, Spain

## Abstract

PARP inhibition (PARPi) has anti-tumor activity against castration-resistant prostate cancer (CRPC) with homologous recombination repair (HRR) defects. However, mechanisms underlying PARPi resistance are not fully understood. While acquired mutations restoring *BRCA* genes are well documented, their clinical relevance, frequency, and mechanism of generation remain unclear. Moreover, how resistance emerges in *BRCA2* homozygously deleted (HomDel) CRPC is unknown. Evaluating samples from patients with metastatic CRPC treated in the TOPARP-B trial, we identify reversion mutations in most *BRCA2/PALB2*-mutated tumors (79%) by end of treatment. Among reversions mediated by frameshift deletions, 60% are flanked by DNA microhomologies, implicating POLQ-mediated repair. The number of reversions and time of their detection associate with radiological progression-free survival and overall survival (*p* < 0.01). For *BRCA2* HomDels, selection for rare subclones without *BRCA2*-HomDel is observed following PARPi, confirmed by single circulating-tumor-cell genomics, biopsy fluorescence *in situ* hybridization (FISH), and RNA*ish*. These data support the need for restored HRR function in PARPi resistance.

## Graphical abstract

**Graphical abstract:**
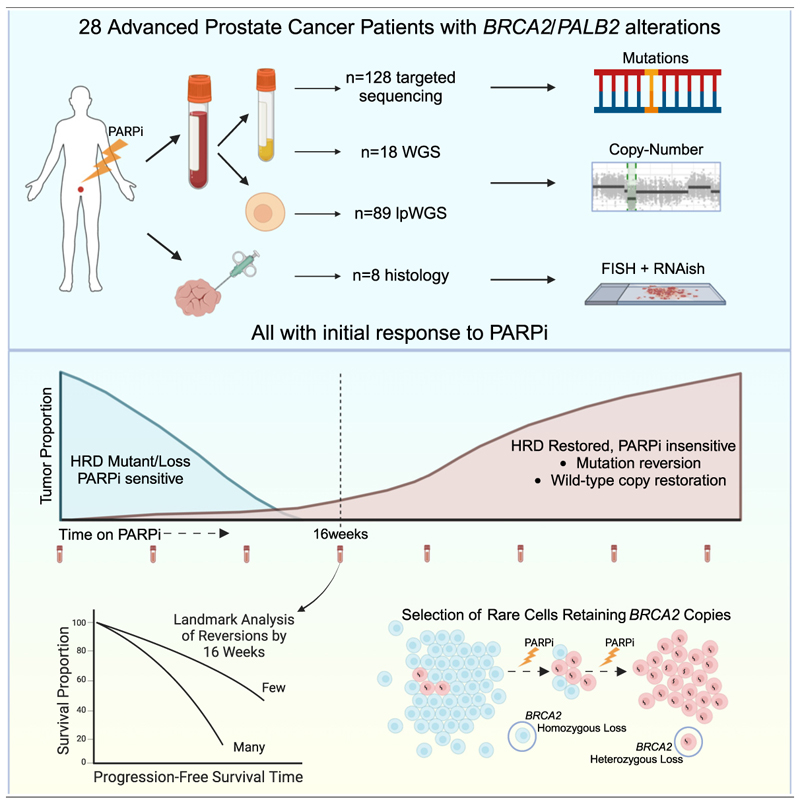

## Introduction

Prostate cancer is the second most diagnosed cancer in men globally and is responsible for significant cancer-related mortality.^1^ The treatment of metastatic castration-resistant prostate cancer (mCRPC) has been transformed with the introduction of poly(ADP-ribose)-polymerase inhibition (PARPi), with phase 3 trials demonstrating survival benefit in mCRPC harboring DNA repair defects including *BRCA2* or *PALB2* mutations.^2–4^ The phase 2 TOPARP-B trial demonstrated the anti-tumor activity of the PARPi olaparib against patients with mCRPC selected for bi-allelic DNA damage repair (DDR) aberrations and that the most durable responses are in tumors with *BRCA2* homozygous deletion.^5,6^ Similarly, the PROFound trial reported that tumors harboring *BRCA2* mutations and heterozygous loss of the other allele have a shorter radiological progression-free survival (rPFS) than those with a *BRCA2* homozygous deletion.^7^

PARPi resistance in tumors with DDR mutations can involve the emergence of reversion mutations in genes including *BRCA2, PALB2*, and other genes involved in homologous recombination repair (HRR), with these mutations restoring some HRR functionality. The clinical relevance of these reversions remains a matter of debate, as does their mechanism of emergence, although DNA polymerase theta (Polθ, POLQ)-mediated microhomology end joining (also known as theta-mediated end joining) has been implicated as a mechanism.^8–11^ Moreover, since homozygous deletions cannot simply acquire reversion mutations, this might explain why patients with castration-resistant prostate cancer (CRPC) with *BRCA2* homozygous deletions have more durable responses to PARPi than tumors with mutations. Whether this is the case or not, the mechanism of PARPi resistance in CRPC with *BRCA2* homozygous deletions remains unknown.

A minimally invasive way to detect tumor genomic alterations is through analysis of circulating tumor DNA (ctDNA); we and others have previously reported *BRCA2* and *PALB2* reversion mutations in the ctDNA of patients with mCRPC at disease progression after response to PARPi.^12–14^ To further characterize the landscape of acquired PARPi resistance in mCRPC, we performed serial ctDNA longitudinal analyses in men whose tumors responded on the TOPARP-B trial and had HRR gene pathogenic mutations, or *BRCA2* homozygous deletions.

## Results

### Patient and sample characteristics

This sub-study comprised 28 patients enrolled on the TOPARP-B trial with a confirmed response to PARPi,^5^ and whose tumors had an alteration in either *BRCA2* or *PALB2*, as these genes have previously been described to revert under treatment pressure. Tumors with pathogenic frameshift or stop-gain mutations were evaluated (*BRCA2 n* = 15, *PALB2 n* = 4), as were tumors bearing homozygous deletions (*BRCA2 n* = 9) (Figure S1). Patient characteristics are described in Table S1.

### Longitudinal detection of reversion mutations

**Figure 1:**
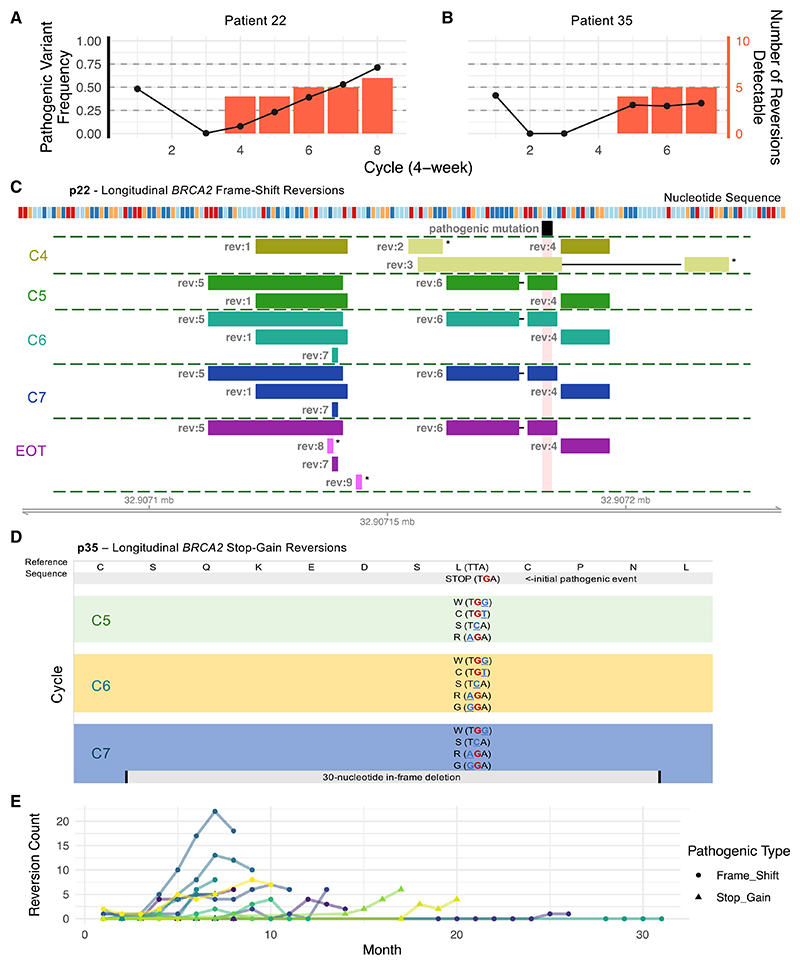
Reversion mutations detectable in the blood of longitudinal mCRPC cfDNA samples (A and B) Example patients (p22 with *BRCA2* frameshift mutation and p35 with *BRCA2* stop-gain mutation) showing changes in somatic pathogenic mutation allele frequency (black) across multiple time points alongside the number of detectable putative reversion variants (red). (C) Schematic of insertion/deletion variants from patient p22 with the capacity to restore reading frame in the context of the pathogenic frameshift variant (black), by time point (C4, cycle 4; C5, cycle 5 etc.; EOT, end of treatment). Bars with asterisks (*) indicate private variants only observed at one time point, other bars are reproduced across multiple time points. Nucleotide sequence shown (dark blue = T, orange = C, red = G, light-blue A). (D) Schematic of alternative codons detectable longitudinally in samples from patient p35 bearing *BRCA2* stop-gain mutation. Amino acids shown along with variant codons in brackets. Initial pathogenic substitution in red, subsequent putative reversion nucleotide substitutions shown in blue. (E) Longitudinal tracking of reversion counts in ctDNA panel sequencing, lines colored by individual patients (*n* = 19, 128 samples). See also Figures S2 and S3.

Targeted next-generation sequencing (NGS) of cell-free DNA (cfDNA) with an average raw coverage depth of ~4,600× was utilized to identify the longitudinal emergence of reversion mutations during PARPi treatment, with at least three samples available for every patient (total *n* = 128 samples from *n* = 19 patients, median *n* = 7 per patient). Putative reversion events impacting *BRCA2* and *PALB2* with the power to restore functional protein were detected using Aardvark.^15^ In the case of pathogenic frameshift mutations, subsequent insertion/deletion events restoring the native reading frame were classified as reversions, while for stop-gains, any nucleotide substitutions that revert a stop codon to a non-stop codon (again restoring a functional reading frame) were considered. Mutations without the capacity to restore a reading frame were not considered. We observed 114 distinct frameshift reversions, split between nucleotide deletions (*n* = 82) and insertions (*n* = 10), and complex events with multiple insertions/deletions (*n* = 22). A further 34 putative stop-gain reversions were detected across 4 patients. Amino acid locations of pathogenic reversions along with reversion counts are shown in Figures S2A and S2B. Among the frameshift deletion-mediated reversions observed in this cohort (*n* = 82), a majority (*n* = 50, 61%) exhibited flanking regions of DNA sequence microhomology with a median length of 2 nucleotides. 84% (*n* = 42) of these microhomology sequences were between 2 and 6 nucleotides, implicating POLQ-mediated end joining (Figure S2C).^16^ Two exemplar patients are presented in Figures 1A–1D, with the original pathogenic mutation allele frequencies regressing to undetectable levels during initial drug response (Figures 1A and 1B) following treatment, and subsequently reappearing alongside novel reversion mutations (Figures 1C and 1D).

Overall, a median of 6 unique reversions were observed per patient, with a striking increase over time; 8/19 patients ended treatment with multiple co-existing reversions (Figure 1E). Reversion variants were usually positioned very close to the original pathogenic variant (Figure S2D). Surprisingly, in *n* = 4 cases, a putative reversion was detected at the baseline time point, and in two cases, it was subsequently seen again at later time points; however, allele frequencies did not rise past 1% (Figure S3A).

Furthermore, almost a third of individuals (*n* = 6) exhibited falls in the number of distinct reversions detected at end of treatment (EOT) (Figure 1E) (sample usually taken some weeks after stopping olaparib), despite an increased proportion of reads assigned to reversions (Figure S3B). This was accompanied with a decrease in the Shannon diversity index at EOT (Figure S3C).

### Emergence of reversion mutations predicts early tumor progression

**Figure 2:**
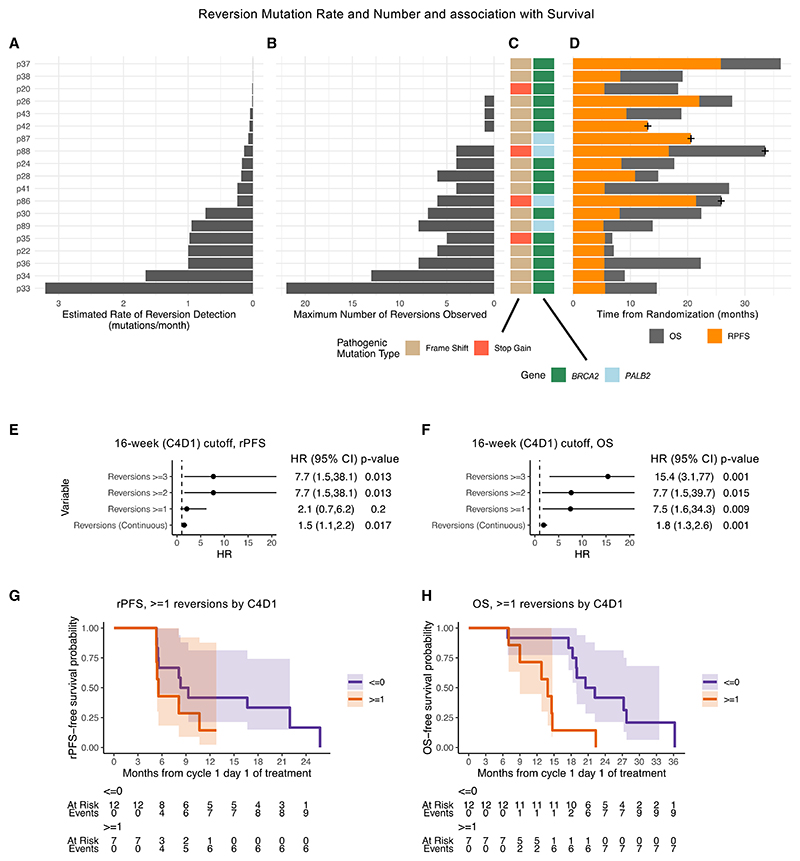
Statistical modeling of emerging reversion count and survival (A) Bar plot of estimated rate of reversion derived from linear regression slope line. (B) Maximum number of reversions observed across all studied time points. (C) Color bar indicating mutation details, red = stop-gains, tan = frameshift, pale blue = germline, mid green = somatic. (D) Swimmer plot of survival time including both radiological progression-free survival (rPFS) (orange) and overall survival (OS) (dark gray), censoring shown with “+” symbol. (A–D) Each bar represents a different patient. (E) Forest plot of results of univariable rPFS mixed-effect time-varying Cox regression, hazard ratios (HRs) with confidence intervals (CIs), and *p* values (Wald test) shown across multiple mutation count cut-points at 16 weeks (C4D1), all patients (*n* = 19) evaluated for reversions (*n* = 38 samples cycle 4 and earlier). (F) Forest plot of results of univariable OS mixed-effect time-varying Cox regression, HRs with CIs, and *p* values (Wald test) shown across multiple mutation count cut-points at 16 weeks (C4D1), all patients (*n* = 19) evaluated for reversions (*n* = 38 samples cycle 4 and earlier). (G) Kaplan-Meier plots of rPFS split by mutation count ≥4 at C4D1, risk table and confidence intervals shown, all patients (*n* = 19) included. (H) Kaplan-Meier plots of OS split by mutation count ≥4 at C4D1, all patients (*n* = 19) included. See also Figure S4, Table S2, and STAR Methods.

To determine the clinical relevance of emergent reversion events, we first calculated the estimated reversion mutation detection rate per patient (mutations/month) (Figure 2A) and found it was significantly negatively correlated with rPFS (Kendall’s tau −0.44, *p* = 0.008) and overall survival (OS) (−0.36, *p* = 0.034). The maximal number of reversion variants detected per patient (Figure 2B) was similarly correlated with poorer rPFS (Kendall’s tau −0.42, *p* = 0.021) but not with OS (−0.29, *p* = 0.095). Individuals with stop-gain pathogenic variants (*n* = 4) had a median estimated reversion rate of 0.19 per month, versus a reversion rate of 0.18 in individuals with frameshift variants (*n* = 15) (Figure 2C). Two patients were censored at radiological progression; all others had complete survival data (Figure 2D).

Using Cox mixed-effect time-varying regression, we observed that by the start of the fourth cycle (C4D1), after 16 weeks of olaparib treatment, the presence of detectable reversion variants in cfDNA was associated with shorter rPFS (one or more: hazard ratio [HR] 2.1 [confidence interval (CI) = 0.7–6.2], *p* = 0.2; two or more: HR 7.7 [1.5–38.1], *p* = 0.013; continuous variable: HR 1.5 [1.1, 2.2], *p* = 0.017) (Figure 2E) and OS (one or more: HR 7.5 [1.6–34.3], *p* = 0.009; two or more: HR 7.7 [1.5–39.7], *p* = 0.015; continuous variable: HR 1.8 [1.3, 2.6], *p* = 0.001) (Figure 2F). At this time point, no individuals had been censored or died, and the proportional hazards assumption was not violated. The median survival times, stratifying based on one or more reversions observed by C4D1, were 8.81 versus 5.59 for rRPFS and 21.4 versus 13.9 for OS (less reversions versus more reversions, respectively) (Figures 2G and 2H). Kaplan-Meier plots of alternative mutation thresholds are shown in Figures S4A–S4D and expanded model results are shown in Table S2.

As tumor fraction is a key consideration in studies of cfDNA in cancer, we sought to evaluate its possible impact as a confounding factor in our findings. The maximum number of reversions detected in cfDNA did not correlate with baseline tumor fraction from whole-genome sequencing (WGS) (Pearson r^2^ = 0.14, *p* = 0.12), suggesting that tumor burden is not closely linked to the capacity of a tumor to develop subsequent reversions (Figure S4E). Furthermore, we performed low-pass WGS (lpWGS) to study tumor fraction in the on-treatment samples at cycle 4, and the presence of tumor fraction >5% was not prognostic for rPFS (HR 1.81 [CI 0.66–5.00] *p* = 0.3) or OS (HR 1.04 [CI 0.35–3.09] *p* = 0.9) at this time point (Figure S4F), nor in a multivariable landmark analysis alongside reversion count, which remained significant (Figure S4G).

## Treatment-induced clearance of *BRCA2* homozygous deletion tumor clones

**Figure 3:**
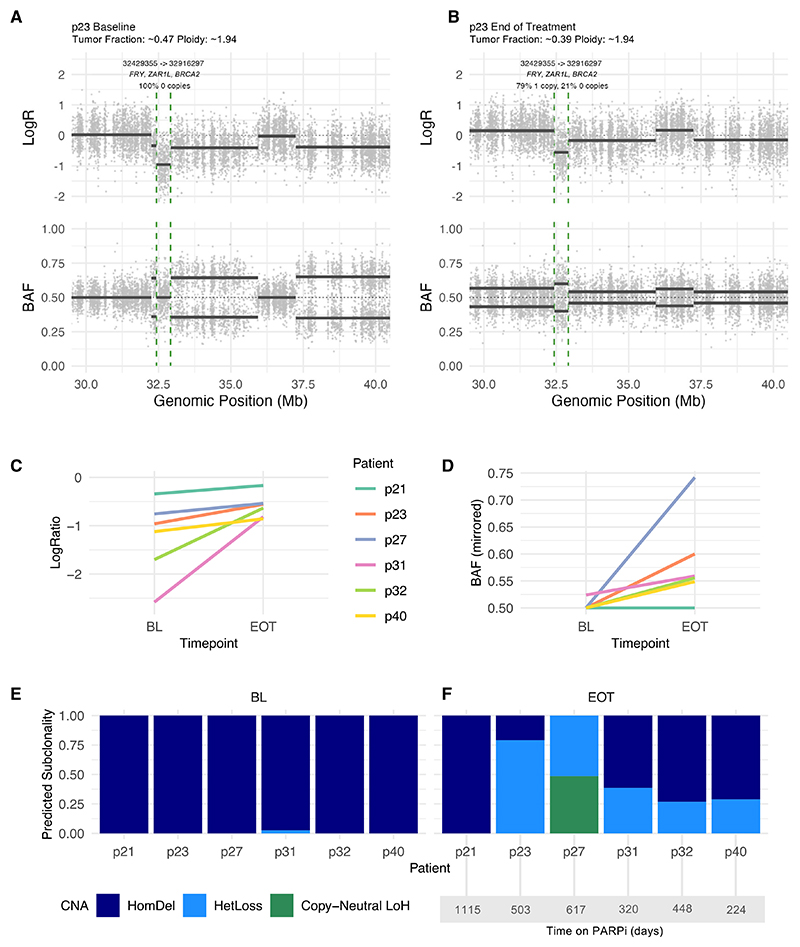
Subclonal shifts observable at the *BRCA2* locus in homozygous-deleted samples through WGS of cfDNA samples (A and B) Example results for patient p23, illustrating changes on chromosome 13. Phased germline B-allele frequency (BAF) and log2ratio (LogR) results for the *BRCA2* locus and surrounding areas shown. Initial homozygous deleted segment indicated using dashed green lines. (C) Changes in log2ratio of *BRCA2*-affecting segment at baseline (BL) and end-of-treatment (EOT). (D) Changes in BAF of *BRCA2* segment pre- and post-PARPi treatment (at BL and EOT). (E and F) Predictions of allele-specific copy-number aberration (CNA) state and associated clonality. All evaluated patients (*n* = 6) bearing a homozygous deletion at baseline could be classified as clonal. By end of study, however, 5 out of 6 showed subclonal events at this locus. Loss of heterozygosity, LoH. See also Figures S5–S8, and Tables S3 and S4.

WGS of 9 responding patients on TOPARP-B, whose tumors had a *BRCA2* homozygous deletion, was performed with an average median coverage of 59× for cfDNA and at 18× for white blood cells (WBCs). The clinical history of these patients is summarized in Table S3. The median tumor fraction across all WGS-sequenced samples was 29% at baseline and 39% at the EOT (Figure S5). The presence, and clonality, of homozygous deletions of *BRCA2* was then explored pre- and post-PARPi therapy. Six patients had evaluable (>10%) tumor purity in cfDNA samples at both baseline and EOT time points (Table S4 and Figure S5). At baseline, all patients had evidence of somatic homozygous deletions of *BRCA2*, as characterized by a negative log2(ratio) value alongside a germline SNP allele frequency of 0.5 (Figure S6). By EOT, dispersion of germline SNP allele frequencies at the *BRCA2* locus was observed following treatment, accompanied by increases in the log2 coverage ratios. An example is shown in Figures 3A and 3B; this change was identifiable across 5 out of 6 patient pairs (Figures 3C and 3D).

The Battenberg algorithm was used to map these values to subclonal copy number states and resolved that baseline samples bore almost entirely clonal *BRCA2* homozygous deletions comprising homozygously deleted tumor cells at baseline (Figure 3E). Surprisingly, following PARPi therapy, cfDNA-predicted clonality indicated a mixture of states and the emergence of subclones without homozygous deletions (Figure 3F). An example is patient p23, depicted in Figure S7, which shows raw allele-frequency and log2(ratio) data and evidence of copy-number shifts specifically impacting chromosome 13 (data for this region across all *BRCA2* HomDel cases are presented in Figure S6B).

A complete homozygous deletion without any identifiable non-homozygous subclones after treatment was observed in one patient (patient p21). This patient was on PARPi treatment the longest, with a deep PSA response and partial radiological response, and displayed evidence of disease oligo-progression. In this case, the EOT sample was obtained 48 days following treatment discontinuation, counter to the other patients from whom it was obtained within 30 days (Table S3).

The presence of various mutational and copy-number signatures^17,18^ was deconvoluted from the WGS ctDNA data at base-line and EOT. This revealed that single-base signatures 3 and 8, classically associated with homologous repair,^19–21^ were present in most cases at baseline and that proportions did not change significantly by EOT (Figure S8). Copy-number signature 3, previously associated with impaired homologous repair,^18^ was present in all *BRCA2* HomDel cases with the proportion declining significantly at EOT (*p* = 0.044, Figure S8), supporting the observation of subclonal selection against HomDel clones.

### Orthogonal studies of *BRCA2* within homozygously deleted tumors

**Figure 4:**
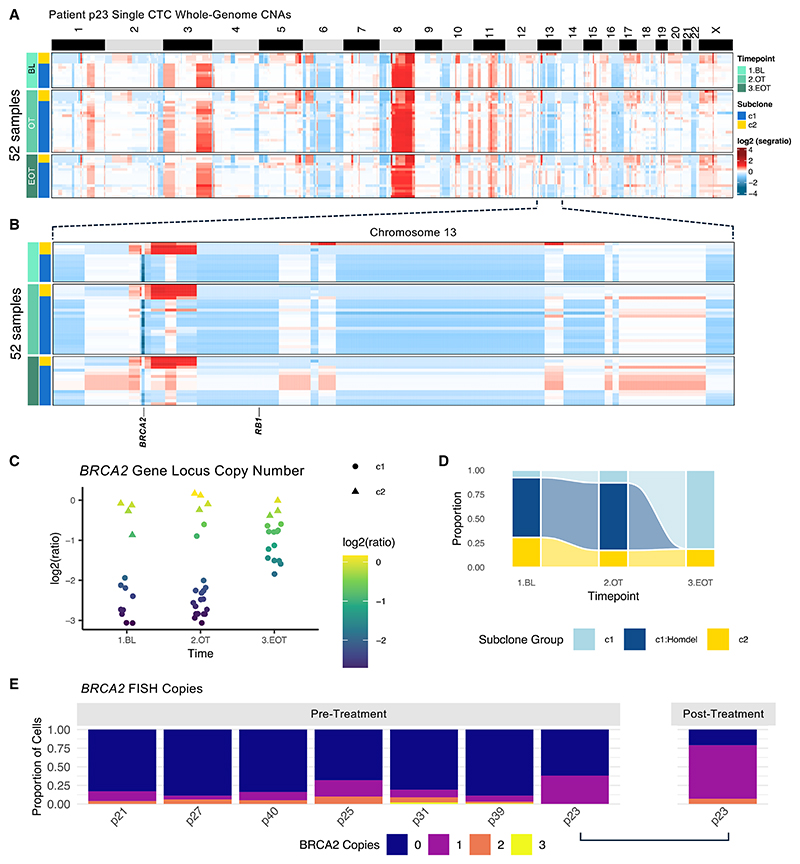
Identification of PARPi-induced longitudinal selection against *BRCA2* homozygous-deleted clones through single-cell assays (A) Genome-wide copy-number aberration (CNA) heatmap of patient p23 low-pass whole-genome sequencing (lpWGS). Each row relates to one cell, each column a genomic position. Chromosomes indicated with black/white bar. Colors mapped to segment log2(coverage ratio). Rows grouped by collection time point (baseline, BL; on-treatment, OT; and end-of-treatment, EOT) and subsequently clustered with hierarchical clustering. Baseline, on-treatment, and end-of-treatment time points are marked, along with major subclone cluster. (B) Zoomed chromosome 13 copy-number heatmap, rows again grouped by collection time point and clustered. (C) Dot plot of *BRCA2* locus showing segment log2(coverage ratio) values. Point shape indicates cells belonging to one of two major clones (c1, clone 1; c2, clone 2). (D) Proportion of circulating tumor cells (CTCs) at different time points split by proposed subclone cluster and the presence of a deep deletion at the *BRCA2* locus (segment log2 ratio < −2), showing longitudinal changes in clone inclusion across time points. (E) Stacked barplots of *BRCA2* FISH copies of pre- and post-treatment biopsy samples, tallied across 100 cells per sample, in individuals bearing homozygous deletions, pre- and post-treatment. Dark blue indicates complete loss; other colors indicate tumor cells bearing copies of *BRCA2*. See also Figures S4, S9, and S10.

To further explore *BRCA2* clonality, lpWGS was performed on 89 single circulating tumor cells (CTCs) and 8 WBCs with an average mean coverage of 0.21× from patient p23, with 52 single CTCs passing filters. Single CTCs closely matched the copy-number profile of the bulk cfDNA data (Figures 4A and S9). Two major subclones were identifiable, with only one bearing a homozygous deletion at the *BRCA2* locus. Striking changes across chromosome 13 in the *BRCA2* homozygous-deleted clone following PARPi treatment were observed (Figure 4B), including the emergence of several low-level gains and a marked increase in the log2(ratio) of the *BRCA2* locus. This supported the appearance of *BRCA2*-competent cells (Figure 4C), and the complete clearance of cells bearing deletions (Figure 4D).

We next hypothesized that rare subclonal *BRCA2* wild-type tumor cells were present prior to treatment with PARPi, persisting in tumor cell populations and providing a pool of resistant DNA repair-competent cells selected for by subsequent treatment. To detect such cells, fluorescence *in situ* hybridization (FISH) and RNA*ish* analyzes were performed on pre-treatment tumor biopsies from patients with *BRCA2* homozygous deletions (Figure 4E). We observed rare cells without *BRCA2* homozygous deletion adjacent to *BRCA2*-deleted cells pre-treatment, and these cells increased in number following PARPi (Figure S10). In all pre-treatment cases with evaluable tissue FISH (*n* = 7 baseline tissue samples, *n* = 1 EOT) (Figure S4), we were able to detect rare tumor cells bearing one or more copies of *BRCA2* (Figures 4E and S10A), with corresponding results for *BRCA2*/centromere ratio (Figure S10B). Using RNA*ish*, we also detected rare cells expressing *BRCA2* mRNA transcripts in four out of six evaluated samples (Figure S10C).

For patient p23, an EOT tissue sample was available. Analysis with FISH and RNA*ish* in this case revealed increases in *BRCA2* gene copy number (proportion with zero *BRCA2* copies at baseline 52%–21% at EOT) and mRNA transcripts (proportion with zero *BRCA2* mRNA transcripts at baseline 73%–35% at EOT) following PARPi treatment, respectively, in line with CTC and ctDNA data (Figures 4E, S10B, and S10C).

## Discussion

PARP inhibition is now a standard of care for treating HRR-defective tumors including advanced prostate cancers with biallelic *BRCA2* and *PALB2* loss, with this improving OS and quality of life.^2,3,7^ Elucidating mechanisms of PARPi resistance can guide the development of next-generation therapeutic strategies for these subjects. Herein, we demonstrate the utility of longitudinal, serial, and non-invasive monitoring of somatic mutations in plasma cfDNA as a tool to understand cancer evolution during olaparib-induced selection pressures in patients with advanced prostate cancer. *BRCA* reversions have been previously associated with PARPi resistance in multiple cancers^14,22,23^; however, serial longitudinal sequencing during PARPi treatment and its association with clinical outcomes and POLQ-mediated microhomology-mediated end joining has been lacking, as have investigations of acquired resistance for tumors with *BRCA2* homozygous deletions.

We now demonstrate a clear link between reversion mutation emergence during PARP inhibition and shorter survival times in mCRPC, with an analysis at the start of the fourth cycle demonstrating poorer rPFS and OS when *BRCA2* or *PALB2* reversions were detected despite initial drug responses. Reversion mutations initially appeared at very low variant allele frequencies. Moreover, at EOT, only a subset of patients displayed high (>20% allele frequency) proportions of reversion reads with a significant subset of patients having very low total reversion allele frequency despite disease progression, with some patients having no reversions detectable. This indicates that either very small reverted subclones are vitally important in disease progression, or that alternative mechanisms of olaparib resistance other than reversions play an important clinical role. Our findings are conceptually supported by studies of bacterial resistance, describing that genetic reversion is nearly always the main form of phenotypic reversion when mutation supply is high (as in cancer).^24^ Numerous studies have identified that a high tumor fraction in cfDNA is overall associated with poor prognosis, but our data here suggest that among responding patients, this effect is minimal when evaluated during treatment, with the detection of *BRCA2/PALB2* reversions offering a greater insight into which patients are rapidly progressing on PARPi therapy.

Interestingly, four patients had putative reversions detectable at baseline before olaparib treatment that were (in two cases) detectable at subsequent time points. These patients had not previously received platinum-based therapies or PARPi; however, two did receive radiotherapy, and as *BRCA2* defects cause radiosensitivity,^25,26^ this could be one explanation for the occurrence of these reversions. Further studies will be needed to validate and determine the relevance of these rare events.

Notably, a majority (61%) of the frameshift deletion *BRCA2* and *PALB2* mutation reversions had an associated DNA microhomology sequence, in line with a pan-cancer analysis of *BRCA* reversion.^10^ These associated microhomology sequences suggest the activity of some form of microhomology-mediated end joining (MMEJ), such as that driven by POLQ, in HRR-defective tumors.^27^ MMEJ can be blocked by POLQ inhibition,^11,28^ and these findings support clinical testing of the hypothesis that the blockade of reversion mutations, and thus therapeutic resistance, can be achieved by combined PARP/POLQ inhibition.^29^ Nevertheless, not all reversions in our cohort were associated with microhomology, suggesting that other processes are at work, and future studies to test DNA repair processes are needed to evaluate whether POLQ is the main driver of microhomology-associated reversions.

While reversion mutations have been described in the context of *BRCA2 or PALB2*-mutant CRPC, it has been unclear what mechanism was driving resistance in tumors with a *BRCA2* homozygous deletion. Since the *BRCA2* gene is completely lost in these tumors, the occurrence of a reversion mutation restoring gene function is highly unlikely. Herein, we demonstrate that these tumors appear to usually restore *BRCA2* function through selection of rare subclones without bi-allelic *BRCA2* deletion. In single-cell analyzes, these were detectable prior to treatment and emerged to become the predominant clone at disease progression. The presence of rare subclones without *BRCA2* homozygous deletion prior to treatment was confirmed by FISH and RNA*ish* on tumor biopsies. These data demonstrate the essential role that HRR function plays in the anti-tumor activity of PARPi since all but one of the patients with mCRPC with *BRCA2* homozygous deletion had, after initial response to PARPi, copy-number data suggesting the presence of a *BRCA2* hemizygous deletion at disease progression. Critically, our data indicate that platinum therapy is not indicated for most patients progressing on PARP inhibition with olaparib since these prostate cancers restore homologous recombination repair.

Strengths of the current analyzes are the presence of many longitudinal, serially taken plasma samples per patient that could be interrogated with targeted NGS for putative reversions, allowing survival modeling, and the use of ctDNA WGS to interrogate *BRCA2* clonality and subsequent correlations with orthogonal methods using CTCs and tumor biopsies.

Overall, our study highlights the complexity of the evolution of resistance to PARPi therapy in prostate cancer and emphasizes the opportunity of ctDNA to improve patient outcomes by enabling the precise and real-time monitoring of patients.

### Limitations of the study

Weaknesses of these analyzes include limited availability of EOT tissue samples, which precludes a comprehensive study of HRR gene function in all patients at progression, and the use of a targeted sequencing panel that may limit the detection of reversions including intronic regions. Furthermore, the overall cohort studied here was modest in size, with only 25 patients evaluable for analysis, and the individuals were heavily pre-treated with both chemotherapy and radiotherapy. Our analysis here was limited to individuals with alterations in genes known to generate reversion variants, but other mechanisms of resistance to PARPi have been described and may also be key to mCRPCs.

## Resource Availability

### Lead contact

Further information and requests for resources and reagents should be directed to and will be fulfilled by the lead contact, Johann de Bono (Johann.de-bono@icr.ac.uk).

### Materials availability

This study did not generate new unique reagents.

## Star+Methods

### Key Resources Table

**Table:** 

REAGENT or RESOURCE	SOURCE	IDENTIFIER
Deposited data		
Raw sequencing data files	This study	EGA accession: EGAD50000000407
Intermediate files including copy-number segments and aardvark data output.	This study	Zenodo accession: https://doi.org/10.5281/zenodo.10853381
Analysis pipelines.	This study	https://github.com/seedgeorge/ Acquired-Resistance-Paper
Software and algorithms		
Battenberg (v.2.2.10)	Nik-Zainal etal. (2012) Cell^32^	https://github.com/Wedge-lab/battenberg
ASCAT(v.3.1.0)	Ross et al. (2020) Bioinformatics^33^	https://github.com/VanLoo-lab/ascat/
GATK Best Practices (v.4.2.6.1)	GATK Consortium	broadinstitute/gatk:4.2.6.1
IchorCNA (v.0.1.0)	Adalsteinsson et al. (2017) Nature Communications^37^	https://github.com/broadinstitute/ichorCNA
Aardvark (v.0.34)	Moreno et al. (2023) Bioinformatics^15^	https://github.com/DavidQuigley/aardvark
Copykit (v0.1.2)	Minussi et al. (2022) Cancer Research^35^	https://github.com/navinlabcode/copykit
Other		
*BRCA2* RNA-ISH probe	Advanced Cell Diagnostics, CA, USA	Cat# 401378
Cyclophilin B – PPIB probe	Advanced Cell Diagnostics, CA, USA	Cat# 313908
*BRCA2* FISH probe	Abnova, Taipei, Taiwan	Cat# FG0135

## Experimental Model and Study Participant Details

### Study design and patient cohort

TOPARP-B was an open-label, multicenter, investigator-initiated, randomized phase 2 trial as previously described.^5^ Briefly, mCRPC patients were preselected for alterations in DDR genes by next-generation sequencing (NGS) on archival or fresh tumor tissue. Eligible patients needed to be ≥ 18 years old with: histological confirmation of prostate adenocarcinoma, progressive disease at inclusion (defined as PSA progression according to PCWG2, or radiological progression per RECIST 1.1 or in bone by PCWG2), castrate-level testosterone, a WHO performance status of ≤ 2, and adequate organ function. Prior treatment with one taxane was mandatory; two prior lines of taxanes were allowed, but other prior chemotherapy was not. Patients were randomized 1:1 between olaparib 300mg and 400mg twice daily. The primary endpoint of the trial was confirmed response, defined as either radiological objective response (by RECIST 1.1 modified with PCWG2 recommendations), a confirmed PSA decrease of ≥ 50% (PSA50) compared to baseline, or a confirmed conversion of circulating tumor cell (CTC) count from ≥5 CTCs/7.5mL to <5 CTCs/7.5mL by the CellSearch assay. The trial was approved by the London-Surrey Borders Research Ethics Committee (ref. 11/LO/2019). The Royal Marsden NHS Foundation Trust and the Institute of Cancer Research co-sponsored the trial. All patients provided written informed consent. This pre-planned analysis was intended to identify mechanisms of acquired resistance to olaparib, and focused on patients having a confirmed response to PARPi on the TOPARP-B trial (*n* = 43) with *BRCA2* and *PALB2* mutations (*n* = 19), and *BRCA2* homozygous deletion (*n* = 9). Consort diagram and patient characteristics table can be found in supplemental materials (Figure S1 and Table S1).

## Method Details

### Cell-free DNA collection, isolation and sequencing

Plasma for ctDNA analysis was collected at baseline and before each cycle of treatment until progression. An end-of-treatment sample was collected at the time of treatment discontinuation, or approximately 30-day after the last dose of drug. Plasma was processed as previously described.^12^ Initially, DNA Streck tubes were spun at 1800 RCF for 15-min at room temperature, and plasma aliquoted and stored at −80°C for downstream analyzes. Cell-free DNA (cfDNA) was isolated using the QIAsymphony (Qiagen) with a circulating DNA kit (Qiagen) and quantified using the Quant-iT High Sensitivity Picogreen Kit (Invitrogen). Targeted sequencing libraries were constructed using a customized GeneRead DNAseq Mix-*n*-Match v2 panel (Qiagen) on 40ng of cfDNA. This assay uses multiplex polymerase chain reaction (PCR)-based targeted enrichment, covering 6025 amplicons across 113 genes.^30^ Libraries were sequenced using the MiSeq Sequencer (Illumina).

cfDNA whole genome sequencing (cfWGS) libraries were generated with 10 ng of cfDNA from baseline (BL), end-of-treatment (EOT), and white blood cells (WBC) using the NEBNext Ultra FS II DNA kit (New England Biolabs) according to manufacturer’s protocol. Samples were run on the NovaSeq 6000 S4 flowcell (Illumina) using 2× 150bp PE and a 300-cycle kit (Illumina). Low-pass whole-genome sequencing of Cycle 4 cfDNA samples was carried out using a Qiagen QiaSeq FX DNA library kit (Qiagen), and sequenced to a target depth of 0.5× on an Illumina NovaSeq 6000 platform (Illumina, San Diego, CA, USA). All BCL files were converted to FASTQ files with bcl2fastq2 software (v.2.17.1.14, Illumina).

### Targeted panel data processing and detection of reversion variants

To detect insertions and deletions with the capacity to restore functional protein following a pathogenic frameshift variant (as identified in the tumor tissue before enrollment on TOPARP-B (1)), we used the Aardvark software (v.0.34).^15^ We applied the “realign_BAM_from_VCF” function to identify these mutations. An alignment window of +/− 4000 base pairs centered on the position of the pathogenic variant (“–window_size 4000”) was used. A minimum DNA alignment quality of 20 was required (“–min_nt_qual = 20”), and at least 0.9% of the sequences were expected to be realigned by Aardvark (“–min_percent_realigned = 0.9”). For distant realignment, a minimum of 15 nucleotides was selected (“–min_nt_for_distant_realign = 15”). Insertions were permitted in the realignment process by Aardvark (“–allow_insertions_in_realign”). In cases with pathogenic stop-gain mutations, we tallied alternative non-reference, non-stop-gain codons with IGV (v.2.16.2). To reduce false positives, we applied a 3-read minimum filter to initially identify a variant for both reversion classes. Multi-nucleotide frameshift deletions were evaluable for microhomology assessment, and we identified the presence and length of microhomology sequences flanking the deletion site.^10^

### Whole-genome sequencing data processing

Plasma whole-genome sequencing files were analyzed with an in-house Nextflow (v.22.04.0) pipeline implementation of the GATK4 best practices guidelines for genomic data. Initially, “.fastq.gz” files were converted to unmapped.bam files with FastqToSam (v.4.2.6.1) and adapters marked using MarkIlluminaAdapters (v.4.2.6.1). Alignment to the b37 reference genome was performed using bwa-mem (v.0.7.17), before using MergeBamAlignment (v.4.2.6.1) to combine with adapter data. Same-sample.bam files split across multiple lanes were merged using samtools (v.1.11), with duplicates marked using MarkDuplicates (v.4.2.6.1), before sorting with sambamba (v.0.8.2). Finally, BaseRecalibrator (v.4.2.6.1) was used to produce analysis-ready files. Quality metrics were evaluated using Picard CollectWgsMetrics (v.2.23.8) and fastqc (v.0.11.9). Somatic mutation calling was performed using Mutect2 and FilterMutectCalls (v.4.2.6.1) in joint-sample multi-tumor mode to produce single-patient.vcf.gz files.

### Mutation signatures

Deconvolution of mutational signatures on WGS data was performed by first filtering the multi-sample.vcf files for high-quality somatic calls (PASS variants from FilterMutectCalls, WBC depth >10 and tumor depth >10, allele frequency in WBC = = 0) into separate baseline and end-of-treatment datasets, and then retaining variants with an allele frequency greater than 2.5% for mutation signature analysis. We then used the DeconstructSigs R package to map the somatic mutation data onto the COSMIC “SBS_96” set of existing mutation signatures.^17^ We applied a signature cutoff of 0.001 and considered SBS signatures 3 and 8 as HRD-associated. Copy number signatures were called on the WGS segmented copy-number data using the Drews chromosomal instability framework from the CINSignatureQuantification package (v.1.2.0)^18^ under default settings.

### Detection of sub-clonal copy number changes

Somatic copy-number change detection, alongside estimation of tumor ploidy and purity, was performed using the Battenberg (v.2.2.10) algorithm.^31^ For each sample set (baseline and end of treatment cfDNA, with white blood cell germline) we performed a multi-sample Battenberg run (gamma = 10, depth = 10) to generate phased SNP b-allele frequencies (BAFs) and depth (log2(ratio)) values. Individuals with a tumor fraction of >10% (estimated by Battenberg) at both timepoints were included in further analysis.

To leverage the benefit of multiple same-patient tumor samples, we applied a custom post-processing script to jointly segment BAF and log2(ratio) values across baseline and end-of-treatment samples to sensitively resolve shared copy-number breakpoints using the asmultipcf (penalty = 15) function from ASCAT (v.3.1.0).^32^ In cases with different ploidy estimates (once rounded to integers) between BL and EOT samples, we perform an extra ASCAT run to test alternate ploidy solutions in a window around ploidy of the highest goodness-of-fit sample, and if successful, reassign ploidy to the new value. Subsequently we leveraged the call-Subclones Battenberg function to identify potential subclonal segments using the segment BAF and LogR values, and make final allele-specific copy number calls. These final segments were then intersected with gene coordinates (hg19) to identify a result per-gene.

### Single CTC isolation and sequencing

Blood samples were collected in CellSave preservative tubes and kept at room temperature before being processed within 96-h; 7.5mL of blood was processed using the CellSearch System according to the manufacturer’s instructions. Following enumeration, CTC cartridges were stored at 4°C, before transfer into fresh Eppendorf tubes, washed twice with 150 μL of phosphate buffered saline, and fluorescence-activated cell sorted (FACS) (FACS Aria III; 140 Becton, Dickinson and Company) to single CTCs (DAPI^+^, CK^+^,CD45^−^) or WBC (DAPI^+^, CD45^+^, CK^−^).^33^ Sorted cells were whole-genome amplified (WGA) using the GenomePlex Single Cell Whole Genome Amplification Kit (WGA4, Sigma) according to the manufacturer’s instructions. DNA was purified (MinEluteTM PCR Purification Kit (Qiagen)) and quantified using Qubit (Invitrogen) and Tapestation 4200 (Agilent). WGS libraries were constructed using 10ng of WGA single-cell DNA and the NEBNext UltraII DNA Library Prep Kit for Illumina according to manufacturer instruction. Pools containing 96 cells was sequenced on a NovaSeq 6000 S4 flowcell lane.

### Bioinformatic processing of CTCs

Per-cell fastq.gz files were aligned to the GRCh37 hg19 reference genome using bwa-mem (v.0.7.17), with duplicates marked and removed using MarkDuplicates (v4.2.6.1). Single-cell.bam files were subsequerntly processed using the R package copykit (v0.1.2),^34^ using the runVarbin function with default parameters. Cells with %80% reads assigned to genomic bins, or with a read count greater than 100 million or less than 500,000 were excluded. Per-bin read counts of cells were normalized against median values from WBCs. Cells were processed further using the following copykit functions: runVst, runSegmentation (multipcf, gamma = 10) and findAneuploidCells (resolution = 0.2). Non-aneuploid cells were excluded from further analysis, along with cells with an over-dispersion value greater than 0.1 and an absolute median segment log2(ratio) value greater than 0.3. Profiles were segmented and smoothed using knnSmooth (k = 2, multipcf gamma = 10) and outliers removed (resolution = 0.8). CTCs were clustered using run-Umap (n_neighbors = 15) and findClusters (k_subclones = 5).

### cfDNA low-pass whole-genome sequencing data processing

lpWGS data were converted to paired-end reads (bcl2fastq2 v.2.17.1.14) with default settings and subsequently aligned to the human reference genome (GRCh37) using the BWA-MEM (version 0.7.12) algorithm.^35^ Quality control checks were performed using Picard (Broad Institute, Cambridge, MA, USA, version 2.8.1) and FASTQC (Babraham Institute, Babraham, UK, version 0.11.8). Samples were excluded from analysis if the sequencing depth was less than 0.05× or if they failed the FASTQC read quality filter. Aligned reads were quantified using HMMcopy readCounter (v.0.99.0) with the quality filter and interval width set to 20 and 500 kb, respectively.

Read depth data were modeled and the tumor fraction was calculated using ichorCNA (version 0.1.0).^36^ Transition strength parameters were set at –txnE = 0.99999 and –txnStrength = 100000; the maximum copy number (CN) was set to 5 to account for amplifications. The germline DNA fraction (values 0.5, 0.6, 0.7, 0.8, 0.9, 0.95, 0.99), ploidy (initial values 2 and 3), and subclonality were modeled. The default 500-kb reference coverage dataset supplied with ichorCNA was used. Tumor purity values were used as input for Cycle 4 statistical analysis.

### *BRCA2* RNA *in situ* hybridisation (RNA-ISH)

RNA *in situ* hybridization (ISH) detection of BRCA2 expression was performed on 4 μm sections derived from FFPE blocks, with a probe for BRCA2 (Cat. No. 401378, Advanced Cell Diagnostics, CA, USA), using the RNAscope 2.5 LS Reagent Kit-BROWN (Cat. No. 322100, Advanced Cell Diagnostics, Hayward, CA, USA) on a BOND RX platform (Leica, Germany) according to the manufacturer’s protocol. A housekeeping gene, peptidylprolyl isomerase B (cyclophilin B – PPIB) probe (Cat. No. 313908, Advanced Cell Diagnostics, CA, USA), was used as internal-control for mRNA quality per sample. Sections were scanned at 40× on a VS200 Research Slide Scanner (Olympus, Tokyo, Japan). Areas of tissue with a PPIB expression of less than 4 spots/cell were excluded from the analyzes. A pathologist (BG) scored 100 intact tumor cells per sample and quantified the number of discrete RNAish spots.

### *BRCA2* FISH

We optimized a dual-color FISH assay, comprising an approximately 170 kb directly-labelled probe with the fluorophore Texas Red targeting the 13q13.1 locus (BRCA2), and an approximately 550 kb directly labeled probe with the fluorophore FITC targeting the 13q centromere (Catalog #FG0135, Abnova). Tissue sections were stained using the ZytoLight Kit (Z-2028, ZytoVision). Briefly, after de-paraffinization and hydration, slides were boiled in pre-treatment buffer for 20 min, followed by pepsin digestion for 5.5 min. The slides were denatured at 75°C for 10 min and subsequently hybridized at 37°C for a minimum of 4 h. After washing, the slides were mounted with Vectashield mounting medium containing DAPI (H-1200, Vector Laboratories). Signals for both probes were counted and recorded for 100 intact, non-overlapping tumor cell nuclei per sample, identified by nuclear morphology by pathologist (BG).

## Quantification and Statistical Analysis

Kendall’s Tau was used to correlate survival with continuous variables. The coxme (v2.2–18.1) and survival (v3.5-7) R packages to generate time-varying mixed-effect Cox regression models for all 19 patients studied for reversions. For each cycle (C1D1 to C5D1), and across each mutation count cut-point (0 versus 1+, ≤1 versus 2+ etc), we assessed the association of data collected up to that point with subsequent survival outcomes (rPFS and overall survival (OS)). Hazard Ratios with 95% Confidence Intervals and *p*-values are shown in Figures 2E and 2F. Proportional hazards assumption was tested with Schoenfeld’s global test.^37^ Reversion mutation rates for each patient were estimated by fitting a linear mixed-effect model to the entire cohort of patients using the nlme R package (v3.1-164), with a random intercept and random slope for each patient, and then extracting per-patient coefficients. Kaplan meier plots shown in Figures 2G and 2H indicate survival times split by presence of reversion at Cycle 4 (16 weeks). Correlation of baseline tumor fraction and subsequent maximum number of reversions in Figure S4E was performed using Pearson’s correlation coefficient, with R^2^ and *p*-value shown. Univariable survival analysis of Cycle 4 tumor fraction, as estimated by lpWGS, versus OS and rPFS in Figure S4F and multivariable survival analysis including Cycle 4 reversion count in Figure S4G were performed for all 19 patients studied using the survival (v3.5-7) R packages, with hazard ratios, confidence intervals and *p*-values shown.

## Supplementary Material

Supplemental information can be found online at https://doi.org/10.1016/j.ccell.2024.10.015.

Supplementary Items

## Data Availability

Raw sequencing data from this study has been deposited in the EGA (accession number EGA: EGAD50000000407). Intermediate files including Aardvark reports and whole-genome copy-number data are held at Zenodo (accession number https://doi.org/10.5281/zenodo.10853381). Sequence data processing pipelines can be found at the GitHub repository https://github.com/seedgeorge/Acquired-Resistance-Paper.

## Highlights

- Monitoring of PARPi-treated mCRPC detects the emergence of resistant subclones
- By 16 weeks, these associate with disease progression and survival
- *BRCA2/PALB2* reversions in mCRPC present signs of microhomology-mediated repair
- Tumors with *BRCA2* HomDels resist PARPi through the selection of *BRCA2*-competent cells

## In brief

Seed et al. explore a cohort of PARPi-treated advanced prostate cancers, studying plasma cell-free DNA samples for genomic alterations that provide drug resistance. They find multiple mutations emerging in parallel over time and show links to clinical outcome, and evidence of how tumors with *BRCA2* deletions can survive PARPi blockade.
