# Supplementary material for: Elucidating acquired PARP inhibitor resistance in advanced prostate cancer
Source: Cancer Cell. Author manuscript; Available in PMC 2025 Aug 14. (PMC7618010; doi:10.1016/j.ccell.2024.10.015)
Supplement: Supplementary Items [file EMS207307-supplement-Supplementary_Items.zip › 1-s2.0-S1535610824004033-mmc1.pdf]

Cancer Cell, Volume *42*

Supplemental information

Elucidating acquired PARP inhibitor resistance in advanced prostate cancer

George Seed, Nick Beije, Wei Yuan, Claudia Bertan, Jane Goodall, Arian Lundberg, Matthew Tyler, Ines Figueiredo, Rita Pereira, Chloe Baker, Denisa Bogdan, Lewis Gallagher, Jan-Phillipp Cieslik, Semini Greening, Maryou Lambros, Rui Neves, Lorena Magraner-Pardo, Gemma Fowler, Berni Ebbs, Susana Miranda, Penny Flohr, Diletta Bianchini, Pasquale Rescigno, Nuria Porta, Emma Hall, Bora Gurel, Nina Tunariu, Adam Sharp, Stephen Pettit, Nikolas H. Stoecklein, Shahneen Sandhu, David Quigley, Christopher J. Lord, Joaquin Mateo, Suzanne Carreira, and Johann de Bono

Figure S1

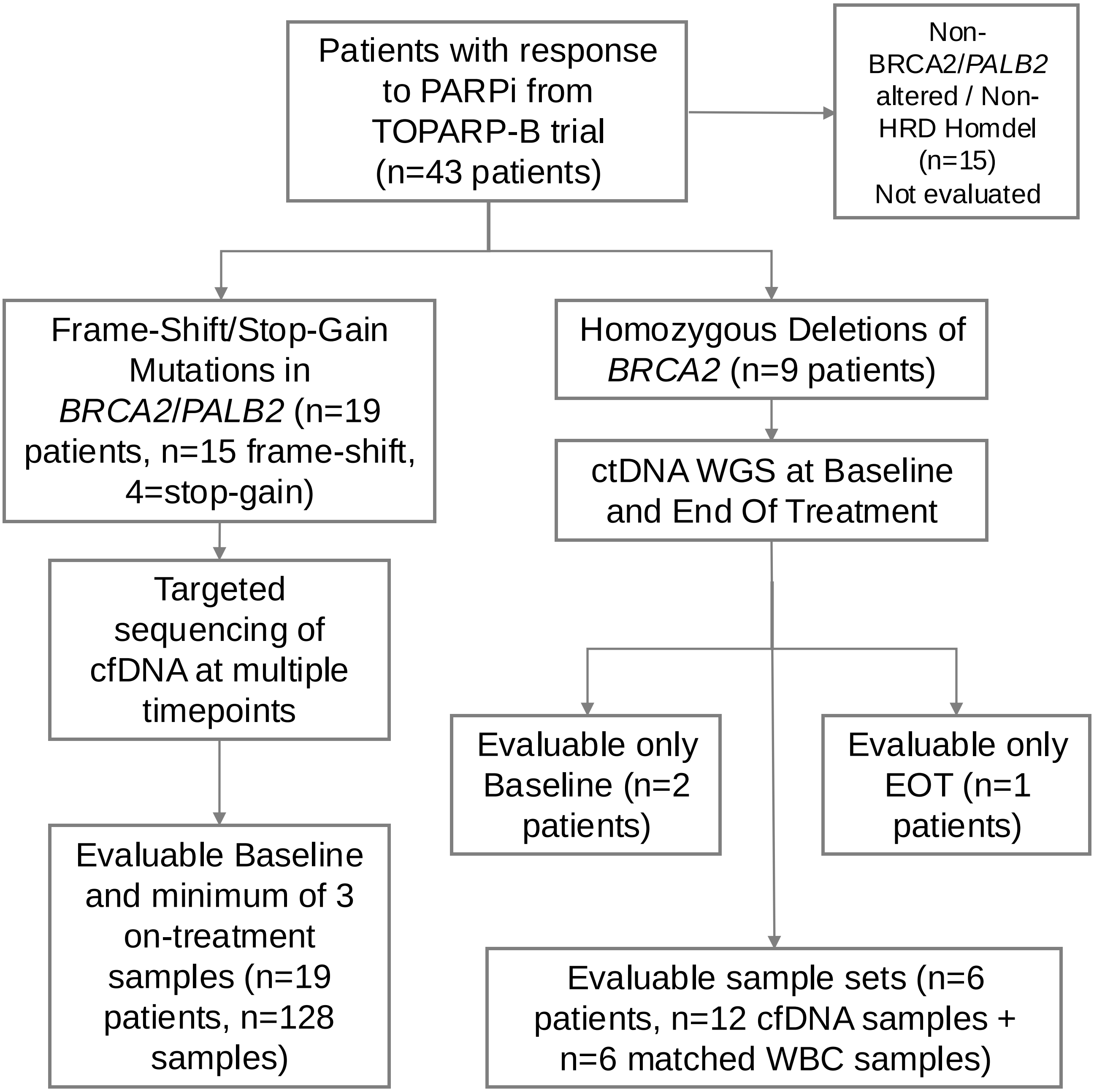

Figure S1: CONSORT diagram of patient cohort included in study, related to STAR Methods. Homdel=Homozygous Deletion, cfDNA=cell-free DNA, ctDNA=circulating tumor DNA, EOT=end-of-treatment, WBC=white blood cell, WGS=whole-genome sequencing.

Figure S2

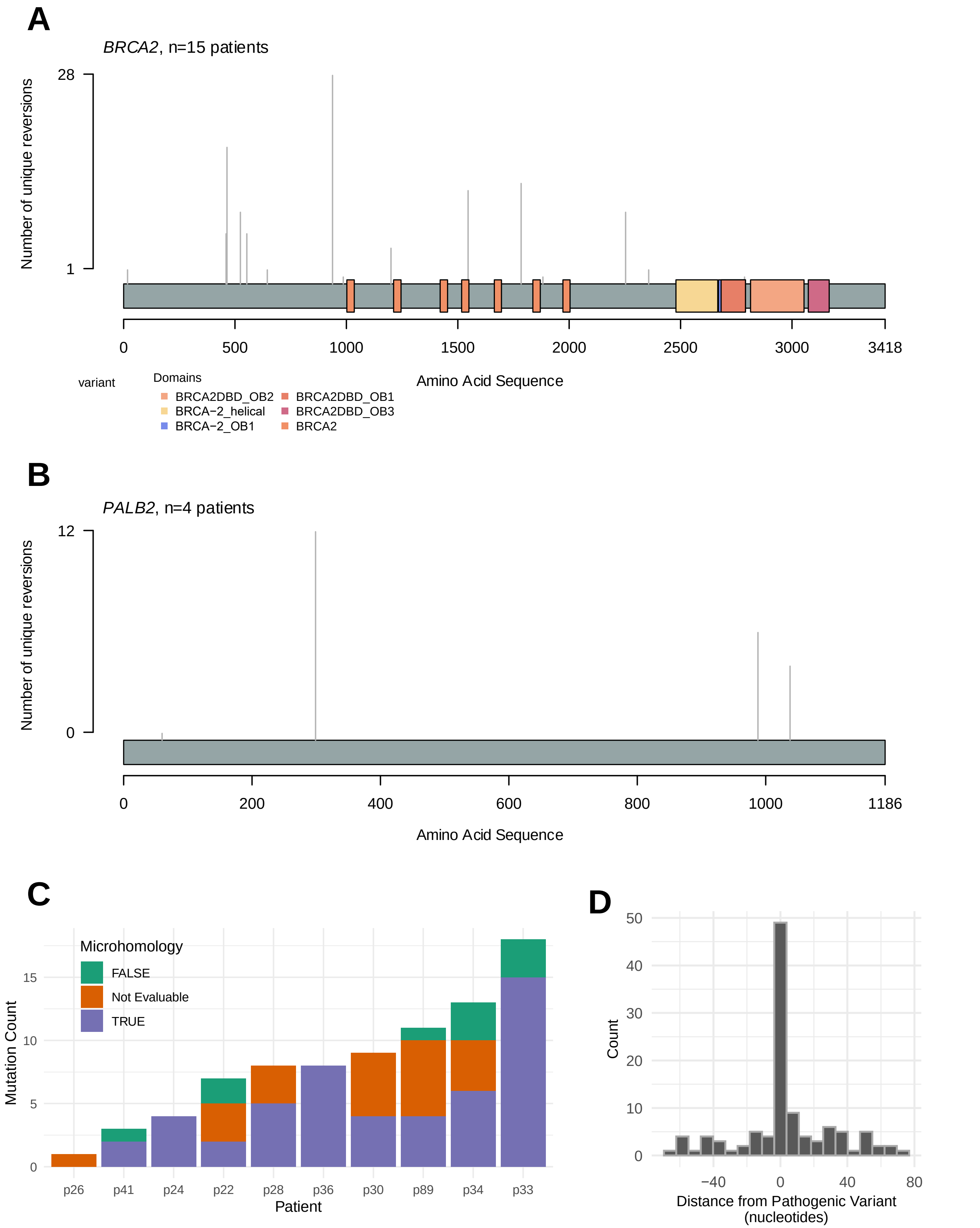

Figure S2: *BRCA2* and *PALB2* reversions, related to Figure 1.
A-B Lollipop plots depicting pathogenic mutations across the amino acid sequence of the *BRCA2* and *PALB2* genes. The height of each line indicates number of unique reversions observed for that variant. Protein domains are indicated with colored sections. **C** Counts of reversions, split by evidence of microhomology among individuals (n=10) bearing nucleotide deletion reversions (n=60 unique variants). Individuals without evaluable frame shift deletion reversions not depicted. **D** Frequency histogram illustrating distance from pathogenic mutation of all evaluated reversion frame-shift variants.

Figure S3

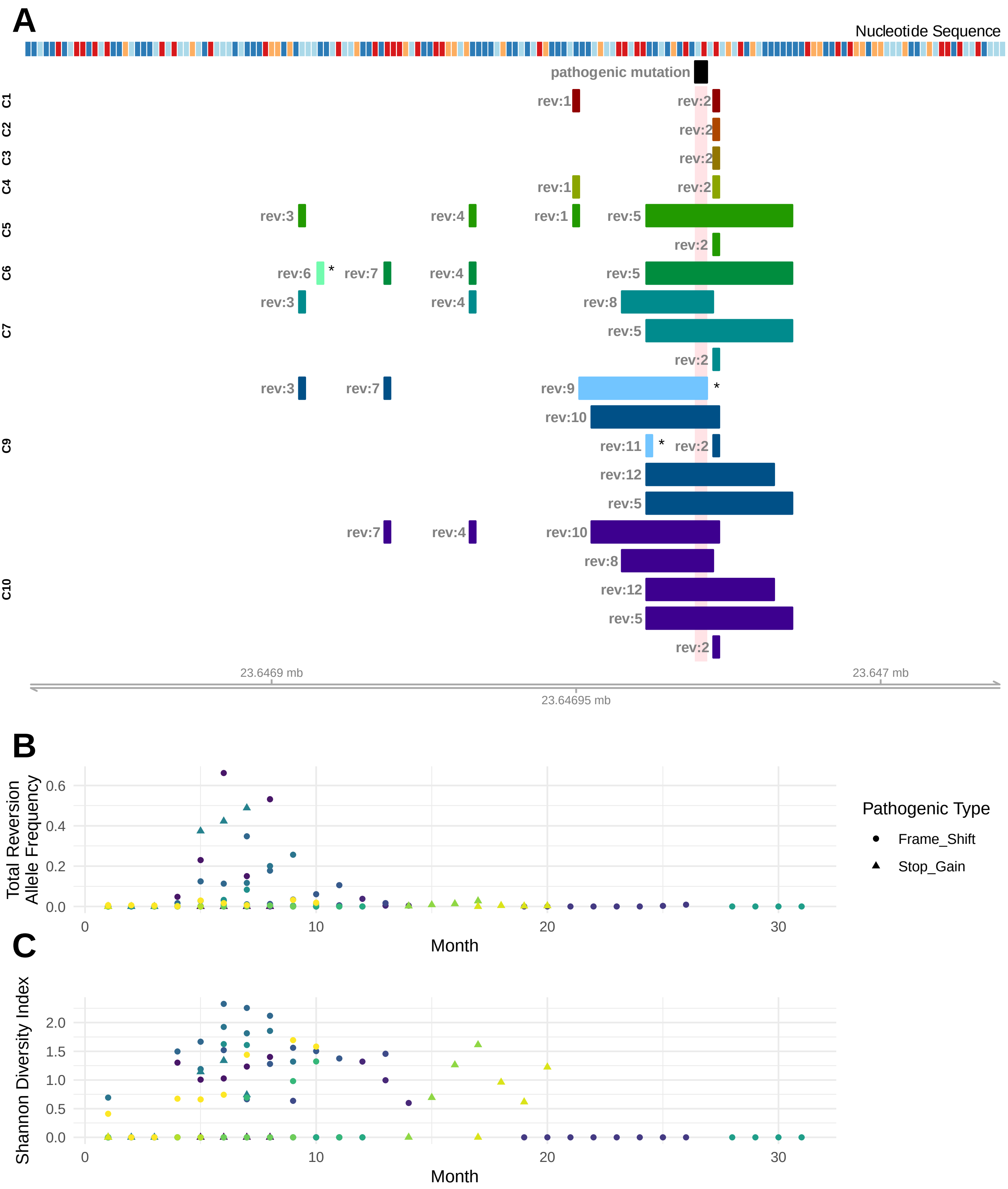

Figure S3: Reversion allele and mutation diversity dynamics, related to Figure 1.
A Example *PALB2* Frame-shift reversions detected in patient p89, with events detected at C1D1. Pathogenic variant shown in black. Nucleotide sequence shown (dark blue=T, orange=C, red=G, light-blue=A). Bars with asterisks (*) indicate private variants observed once, other bars indicate variants found across multiple timepoints. **B** Longitudinal tracking of total reversion allele frequencies in ctDNA panel sequencing, lines colored by patient. **C** Shannon diversity index computed at each timepoint from read counts assigned to each reversion event.

Figure S4

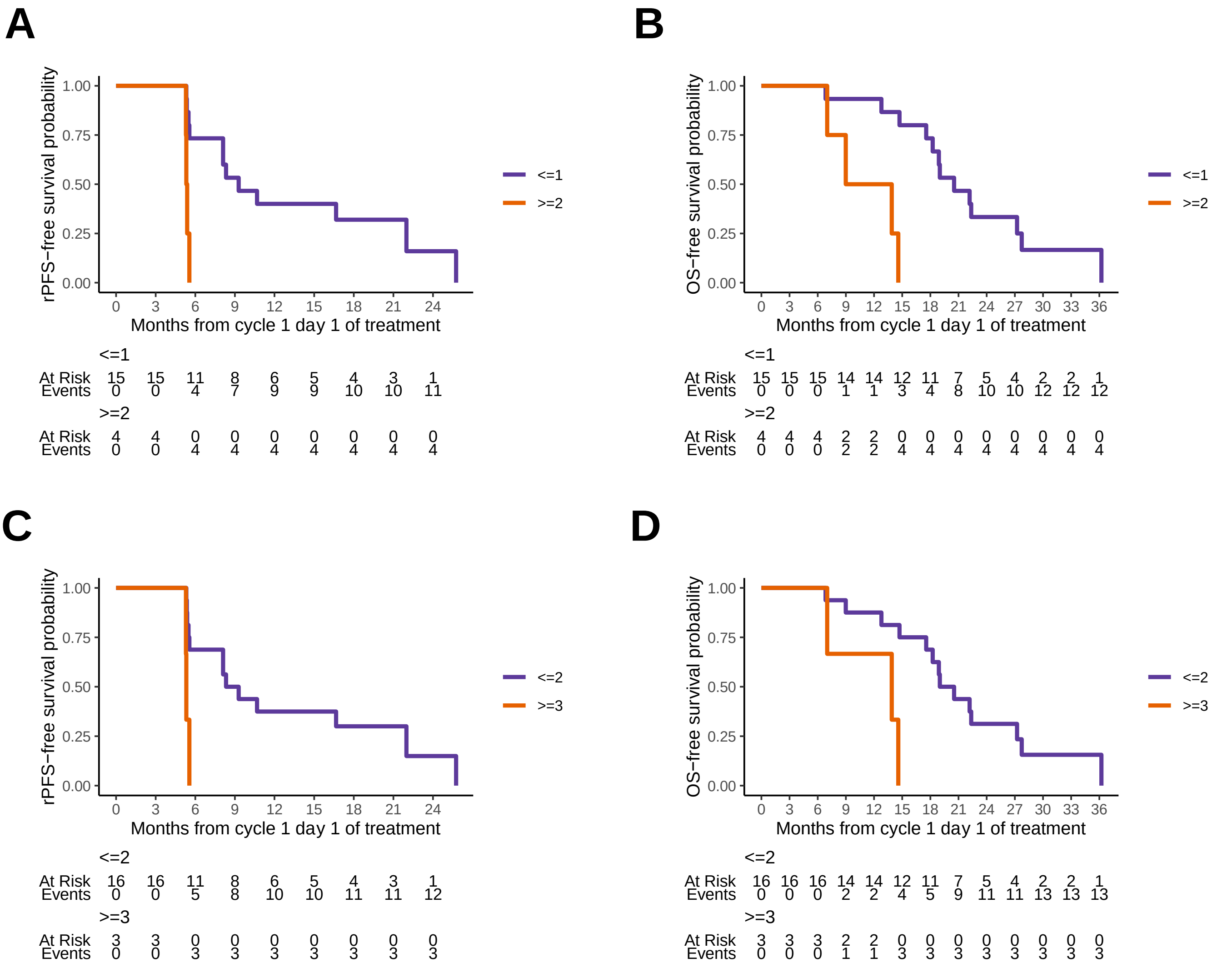

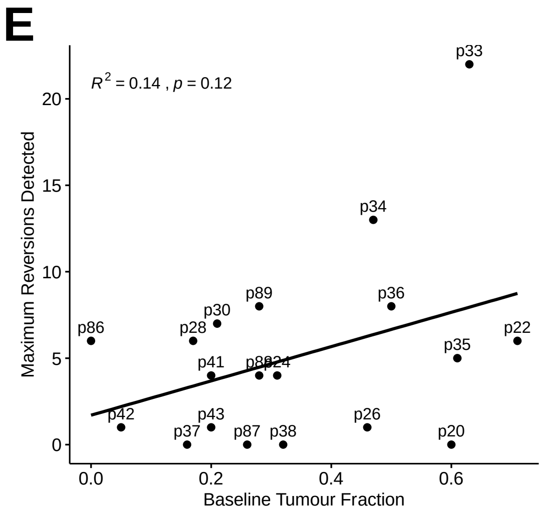

F

Endpoint	Variable	HR	CI	p-value
RPFS	C4 Fraction >=5%	1.04	0.35-3.09	0.9
	C4 Fraction >=10%	2.9	0.74-11.31	0.1
	C4 Fraction Continuous	6.31	0.26-151.45	0.3
OS	C4 Fraction >=5%	1.81	0.66-5.00	0.3
	C4 Fraction >=10%	3.31	0.88-12.38	0.08
	C4 Fraction Continuous	6.46	0.36-115.04	0.2

G

Endpoint	Variable	HR	CI	p-value
rPFS	Reversion Count	1.57	1.08-2.30	p=0.019
	C4 Fraction >=5%	0.64	0.17-2.33	p=0.496
rPFS	Reversion Count	1.53	1.10-2.14	p=0.012
	C4 Fraction >=10%	3.66	0.88-15.25	p=0.075
OS	Reversion Count	1.55	1.09-2.21	p=0.015
	C4 Fraction >=5%	1.43	0.49-4.15	p=0.516
OS	Reversion Count	1.64	1.15-2.34	p=0.007
	C4 Fraction >=10%	3.95	0.99-15.73	p=0.052

Figure S4: Kaplan-meier curves maintain separation across cut points, related to Figure 2 and STAR Methods. **A-B** Kaplan-Meier plots of rPFS (**A**) and OS (**B**) split by mutation count >=2 at C4D1, risk table and confidence intervals shown, survival from trial initiation shown. **C-D** Kaplan-Meier plots of rPFS (**C**) and OS (**D**) split by mutation count >=3 at C4D1, risk table and confidence intervals shown. **E** Pearson correlation of baseline tumor fraction estimate (via WGS) with subsequent maximum number of reversions observed for each sample, pearson test p-value shown with R^2 correlation coefficient. **F** Univariable analysis of Cycle 4 tumor fraction for both OS and rPFS. Hazard ratio (HR), confidence intervals (CI) and p-value (Cox regression Wald test) shown. **G** Multivariable Cox regression models for landmark analysis of Cycle 4 tumor fraction exploring reversion count as a continuous variable alongside varying tumor fraction cutpoints (5% versus 10% for both OS and rPFS. Hazard ratio, confidence intervals and p-value (Wald test) shown.

Figure S5

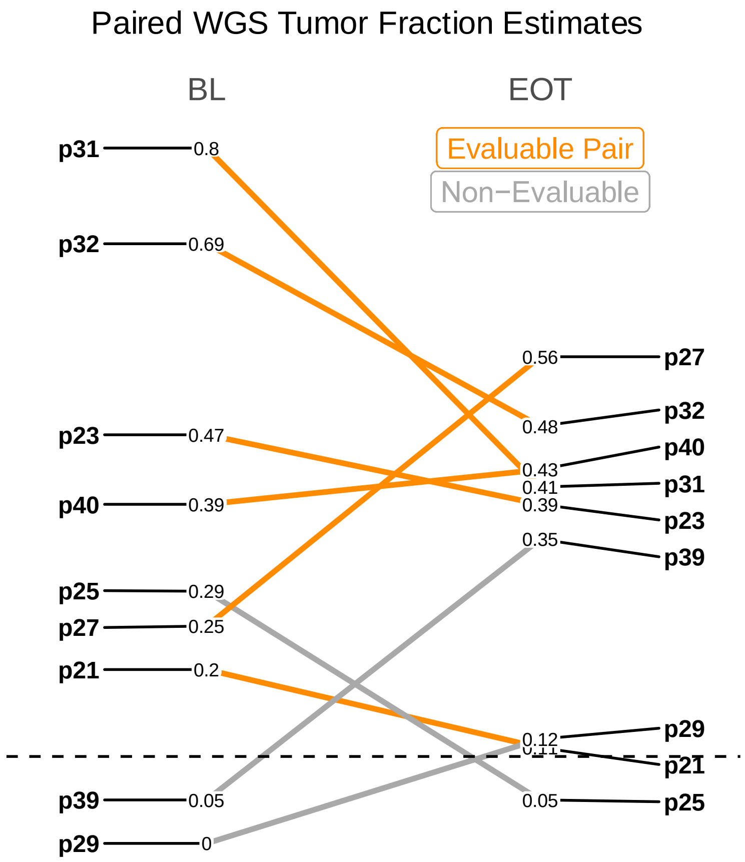

Figure S5: cfDNA WGS sample tumor fraction estimated by Battenberg software between timepoints, related to Figure 3.
Evaluable pairs (both BL and EOT tumor fraction > 0.1) indicated in orange. Dashed line indicates 10% tumor fraction. BL=baseline, EOT=end-of-treatment.

Figure S6

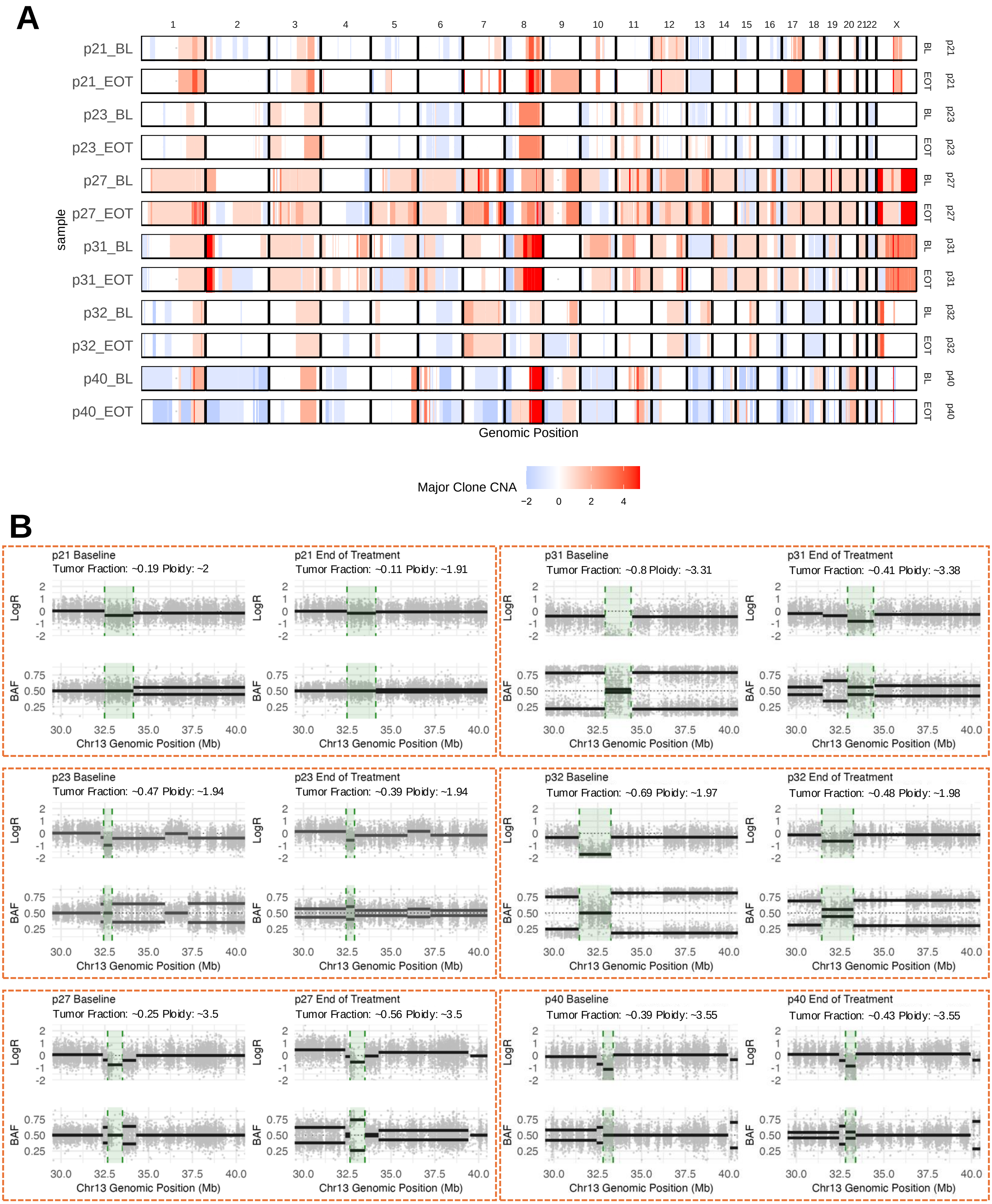

Figure S6: Genome-wide and focal CNAs of *BRCA2*-deleted cases, related to Figure 3.
A Genome-wide heatmap of Copy Number Aberrations (CNAs) across *BRCA2*-deleted tumors, plotting purity and ploidy-adjusted calls for each segment. Major clones depicted in segments with mixed clonal status. BL=Baseline, EOT=End of Treatment. **B** Focal *BRCA2* CNAs on chromosome 13. Phased germline B-Allele Frequency (BAF) and Log2-Ratio results for the 30-40Mb area shown. Initial homozygous deleted segment (spanning the *BRCA2* gene) indicated using dashed green lines and rectangle. Same-patient sets indicated with dashed orange lines.

Figure S7

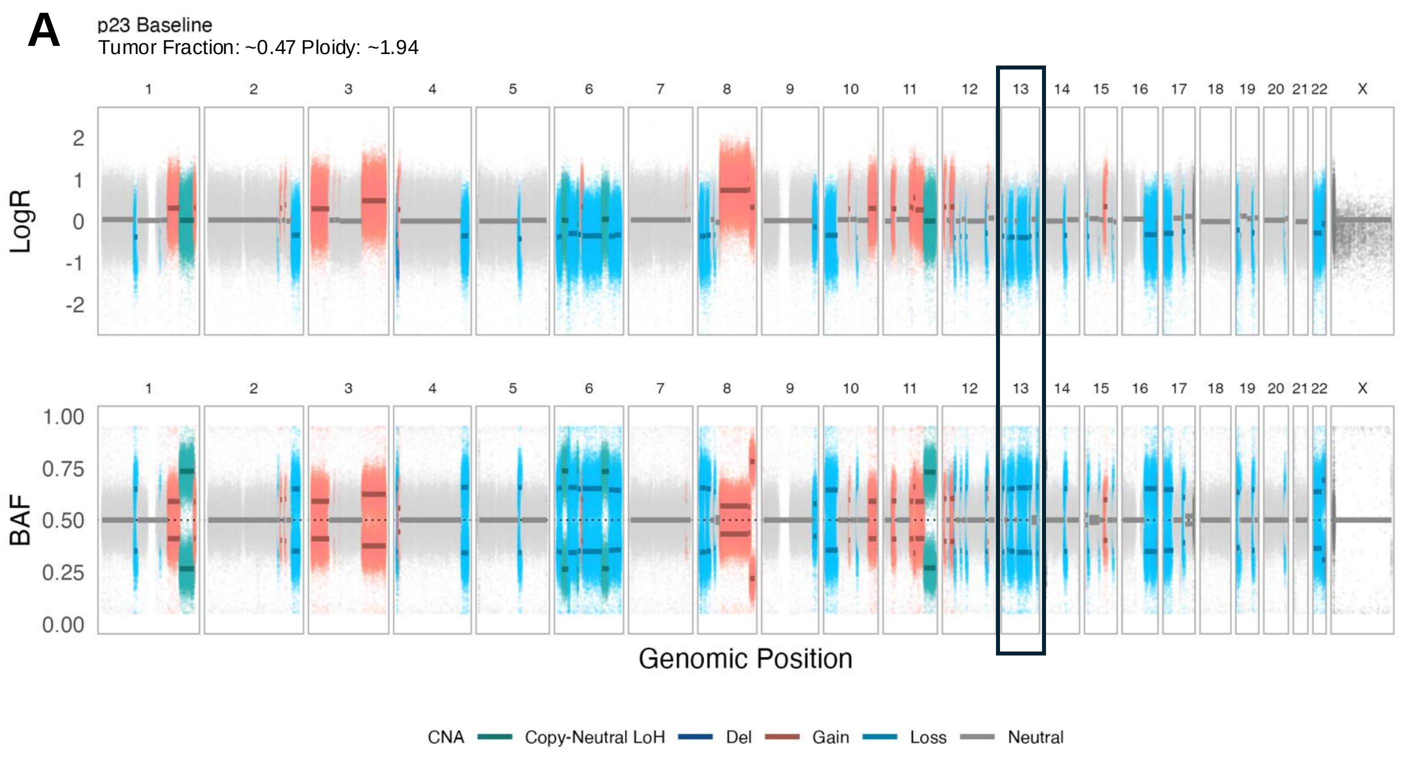

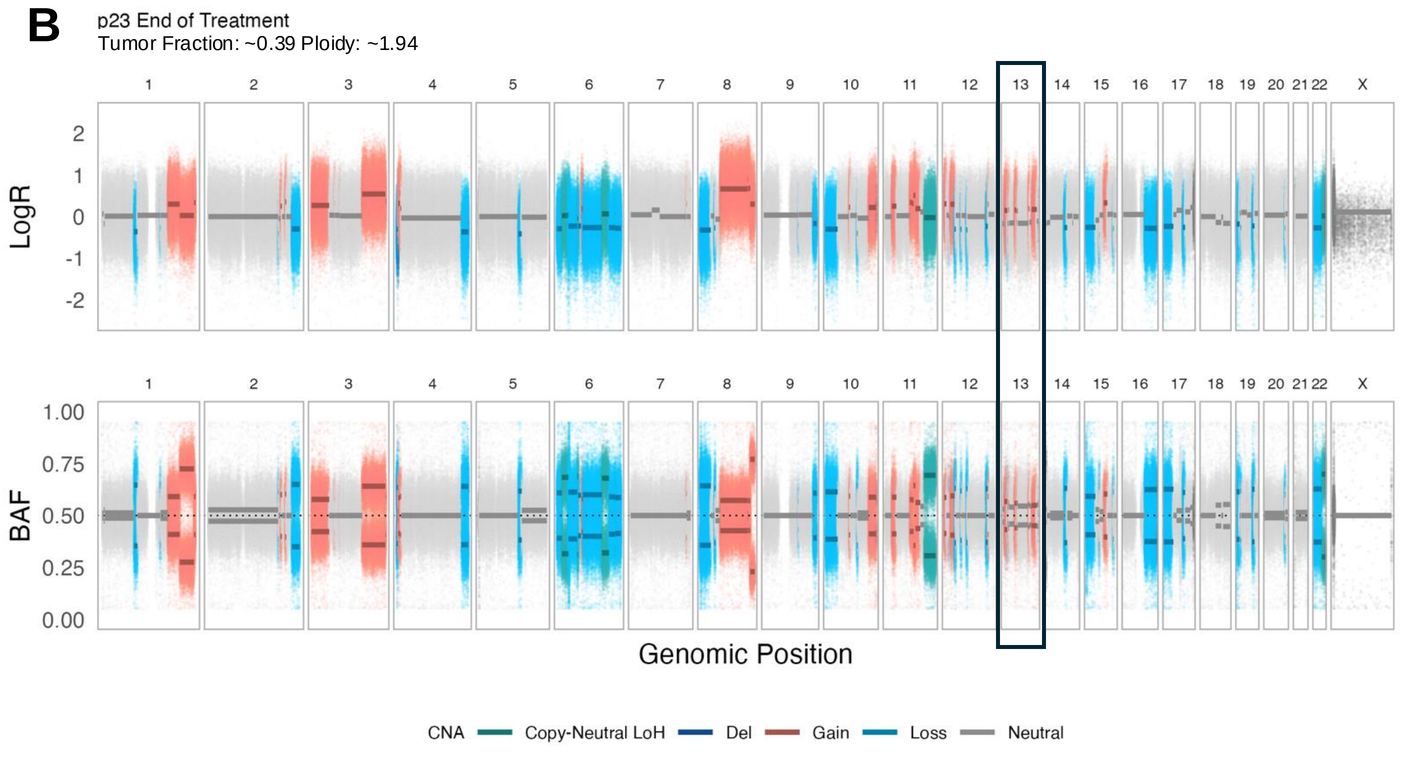

Figure S7: Whole-genome copy-number profiles of p23, related to Figure 3.
A Results for Baseline (pre-PARPi) timepoint and **B** End of Treatment (post-PARPi) samples show broadly similar results, except for chromosome 13. BAF=Phased germline B-Allele Frequency, LogR=Log2Ratio, BL=Baseline, EOT=End of Treatment, Del=Deletion, LoH=Loss of Heterozygosity.

Figure S8

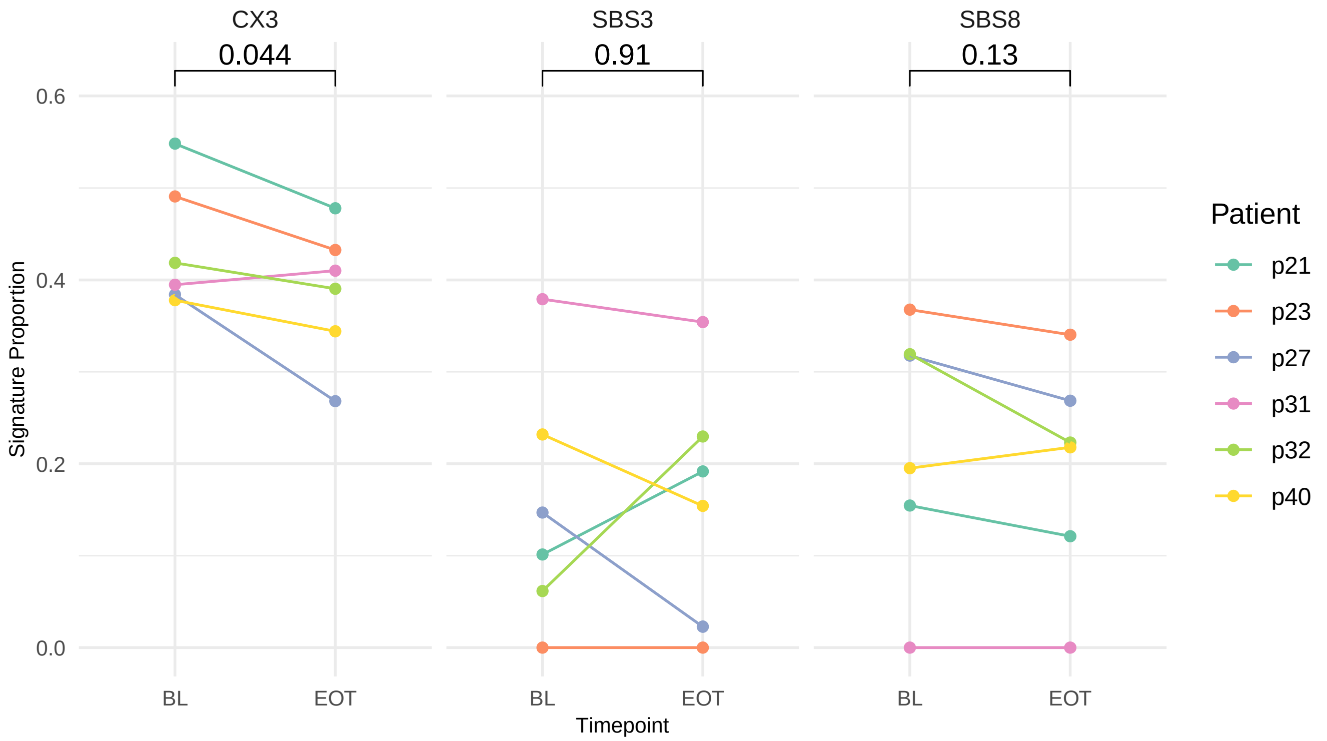

Figure S8: Signature proportions for HRD-associated mutational signatures, related to Figure 3.
Slope graphs for: copy-number signature 3 (CX3), single base signatures 3 and 8 (SBS3 and SBS8) for *BRCA2* homozygous deletion sample set pre- and post-treatment with PARPi**.** Paired two-tailed t-test p-values shown.

Figure S9

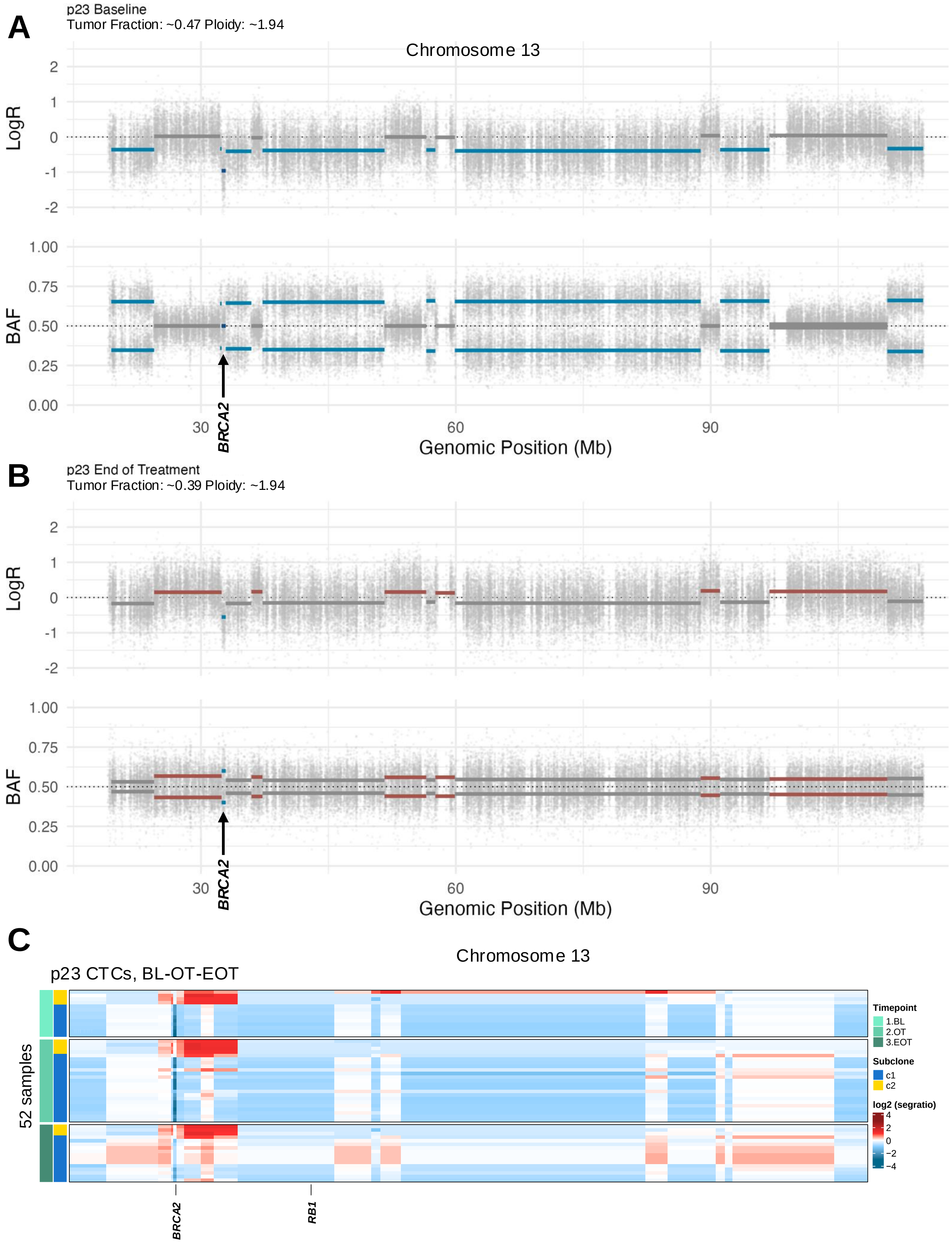

Figure S9: Copy-number events in patient p23, related to Figure 4.
BRCA2 deletion segment detectable in cell-free DNA (cfDNA) whole-genome sequencing (WGS) at **A** baseline**,** and **B** end of treatment, were also observable in **C** circulating tumor cell **(**CTC) lpWGS**.** BAF=Phased germline B-Allele Frequency, LogR=Log2Ratio, BL=Baseline, EOT=End of Treatment.

Figure S10

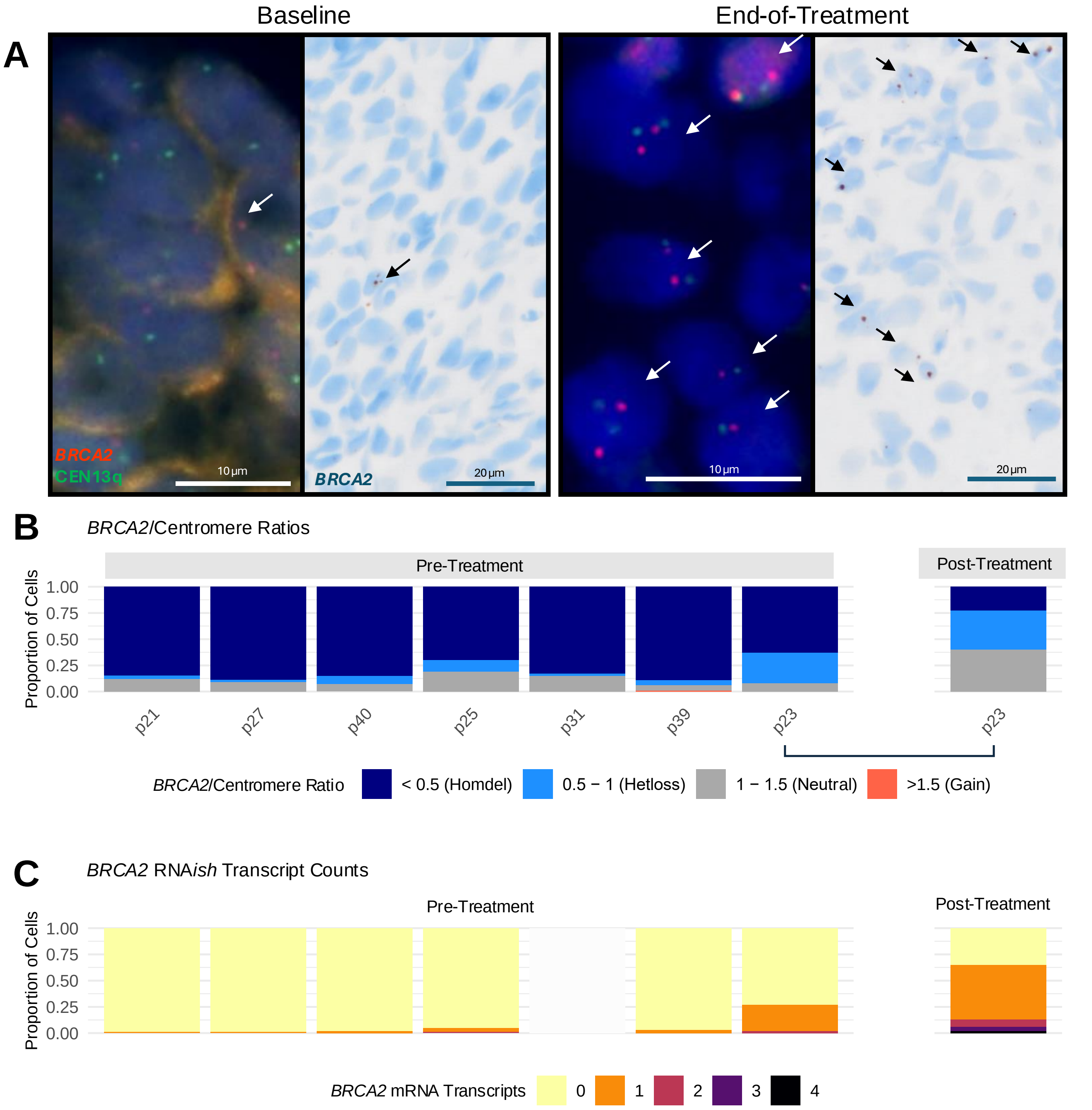

Figure S10: FISH validation of *BRCA2* homozygous deletions, related to Figure 4.
A Example images showing rare but present tumor cells bearing copies of the *BRCA2* gene through FISH and RNA*ish* of biopsies in pre-treatment (BL) and end-of-treatment (EOT) samples. **B** *BRCA2* FISH gene/centromere ratios. **C** Proportion of cells positive for RNA*ish* *BRCA2* transcripts, color indicates number of distinct spots identifiable.

Table S1: Baseline characteristics of all patients evaluated in this sub-study analysis of TOPARP-B trial, related to STAR Methods.

Data are median (IQR) or n (%). Percentages might not add up to 100% due to rounding. RECIST=Response Evaluation Criteria in Solid Tumors, PSA=Prostate Specific Antigen, CTC=Circulating Tumor Cells. * More than one site could be reported. $ One participant in the *BRCA2* homozygous deletion group had lymph nodes metastases marked as "Unobtainable" at trial entry.

	BRCA2/PALB2 mutation (N=19)	*BRCA2* homozygous deletion (N=9)	Overall (N=28)
Age at trial entry	61.1 (57.9, 69.1)	65.9 (64.0, 70.8)	65.5 (58.3, 69.4)
Years from initial diagnosis	3.55 (1.58, 5.82)	6.94 (3.84, 8.10)	3.93 (2.06, 6.76)
Years from diagnosis of castration-resistant prostate cancer	2.36 (0.78, 3.96)	3.55 (2.35, 4.35)	2.41 (1.40, 4.07)
Metastatic disease at diagnosis			
Yes	11 (57.9%)	1 (11.1%)	12 (42.9%)
No	8 (42.1%)	7 (77.8%)	15 (53.6%)
Unobtainable	0 (0%)	1 (11.1%)	1 (3.6%)
Gleason score at diagnosis			
<=7	5 (26.3%)	3 (33.3%)	8 (28.6%)
>=8	12 (63.2%)	5 (55.6%)	17 (60.7%)
Not available	2 (10.5%)	1 (11.1%)	3 (10.7%)
Evidence of progression at trial entry			
PSA progression only	5 (26.3%)	3 (33.3%)	8 (28.6%)
Radiographic progression (with or without PSA progression)	14 (73.7%)	6 (66.7%)	20 (71.4%)
Site of metastatic disease at trial entry* (lung)	3 (15.8%)	1 (11.1%)	4 (14.3%)
Site of metastatic disease at trial entry*$ (lymph nodes)	15 (78.9%)	5 (55.6%)	20 (71.4%)
Site of metastatic disease at trial entry* (liver)	5 (26.3%)	2 (22.2%)	7 (25.0%)
Site of metastatic disease at trial entry* (bone)	15 (78.9%)	8 (88.9%)	23 (82.1%)
PSA at trial entry	49.0 (21.9, 338)	371 (72.0, 474)	118 (24.4, 434)
Prostatectomy	2 (10.5%)	3 (33.3%)	5 (17.9%)
External beam radiotherapy	10 (52.6%)	4 (44.4%)	14 (50.0%)
Radium-223	2 (10.5%)	2 (22.2%)	4 (14.3%)
Biphosphonates	1 (5.3%)	0 (0%)	1 (3.6%)
Docetaxel	19 (100%)	9 (100%)	28 (100%)
Cabazitaxel	6 (31.6%)	4 (44.4%)	10 (35.7%)
Abiraterone acetate	10 (52.6%)	4 (44.4%)	14 (50.0%)
Enzalutamide	8 (42.1%)	6 (66.7%)	14 (50.0%)
Abiraterone acetate or enzalutamide or both	16 (84.2%)	8 (88.9%)	24 (85.7%)
CTC count per 7·5 mL blood at trial entry			
CTC <5	8 (42.1%)	1 (11.1%)	9 (32.1%)
CTC >=5	11 (57.9%)	8 (88.9%)	19 (67.9%)
RECIST 1.1 soft tissue disease			
Measurable disease (with or without bone lesions)	14 (73.7%)	5 (55.6%)	19 (67.9%)
Non-measurable disease (with or without bone lesions)	3 (15.8%)	3 (33.3%)	6 (21.4%)
Bone lesions only	2 (10.5%)	1 (11.1%)	3 (10.7%)

Table S2: Regression model results for time-varying mixed-effect Cox regression model exploring different reversion count thresholds and time cutoffs, related to Figure 2.
Radiographic progression-free survival (rPFS) and overall survival (OS) results shown in separate sub-tables. Hazard ratio (HR) and upper and lower 95% confidence intervals (L95 and U95 respectively) shown. P-value derived from regression model Wald test.

Survival	Variable	HR	L95	U95	p-value	Time_Months	Time_Weeks
OS	Reversions >=1	2.45814115	0.7425383	8.13757	0.14	0.9256198	4
OS	Reversions >=1	2.18132456	0.6466193	7.358545	0.21	1.8512397	8
OS	Reversions >=1	2.16148306	0.6463864	7.227889	0.21	2.7768595	12
OS	Reversions >=1	7.50062418	1.638438	34.337194	0.0094	3.7024793	16
OS	Reversions >=1	6.38631878	1.6039763	25.427475	0.0085	4.6280992	20
OS	Reversions >=2	6.69519483	1.0982233	40.816503	0.039	0.9256198	4
OS	Reversions >=2	1.537E-09	0	Inf	1	1.8512397	8
OS	Reversions >=2	1.433E-09	0	Inf	1	2.7768595	12
OS	Reversions >=2	7.6554465	1.4760388	39.704825	0.015	3.7024793	16
OS	Reversions >=2	18.9303029	3.3875204	105.787221	0.00081	4.6280992	20
OS	Reversions >=3					0.9256198	4
OS	Reversions >=3					1.8512397	8
OS	Reversions >=3					2.7768595	12
OS	Reversions >=3	15.3924032	3.0749748	77.049762	0.00088	3.7024793	16
OS	Reversions >=3	28.7082423	4.7062708	175.120219	0.00027	4.6280992	20
OS	Reversions >=4					0.9256198	4
OS	Reversions >=4					1.8512397	8
OS	Reversions >=4					2.7768595	12
OS	Reversions >=4	15.3924032	3.0749748	77.049762	0.00088	3.7024793	16
OS	Reversions >=4	28.7082423	4.7062708	175.120219	0.00027	4.6280992	20
OS	Reversions (Continuous)	2.33126216	0.9975516	5.448122	0.051	0.9256198	4
OS	Reversions (Continuous)	1.52548367	0.5562323	4.183685	0.41	1.8512397	8
OS	Reversions (Continuous)	1.56478183	0.5609212	4.365216	0.39	2.7768595	12
OS	Reversions (Continuous)	1.82649384	1.2848788	2.596416	0.00079	3.7024793	16
OS	Reversions (Continuous)	1.70814059	1.2641239	2.308116	0.00049	4.6280992	20

Survival	Variable	HR	L95	U95	p-value	Time_Months	Time_Weeks
RPFS	Reversions >=1	2.072702	0.6353447	6.761832	0.23	0.9256198	4
RPFS	Reversions >=1	1.665084	0.4563628	6.075223	0.44	1.8512397	8
RPFS	Reversions >=1	2.078035	0.634895	6.801485	0.23	2.7768595	12
RPFS	Reversions >=1	2.066179	0.6870537	6.213628	0.2	3.7024793	16
RPFS	Reversions >=1	8.797094	2.0585684	37.593532	0.0033	4.6280992	20
RPFS	Reversions >=2	8.523918	1.4044889	51.73211	0.02	0.9256198	4
RPFS	Reversions >=2					1.8512397	8
RPFS	Reversions >=2					2.7768595	12
RPFS	Reversions >=2	7.683036	1.5496698	38.091367	0.013	3.7024793	16
RPFS	Reversions >=2	6.304917	1.6657686	23.864042	0.0067	4.6280992	20
RPFS	Reversions >=3					0.9256198	4
RPFS	Reversions >=3					1.8512397	8
RPFS	Reversions >=3					2.7768595	12
RPFS	Reversions >=3	7.683036	1.5496698	38.091367	0.013	3.7024793	16
RPFS	Reversions >=3	6.304917	1.6657686	23.864042	0.0067	4.6280992	20
RPFS	Reversions >=4					0.9256198	4
RPFS	Reversions >=4					1.8512397	8
RPFS	Reversions >=4					2.7768595	12
RPFS	Reversions >=4	7.683036	1.5496698	38.091367	0.013	3.7024793	16
RPFS	Reversions >=4	6.304917	1.6657686	23.864042	0.0067	4.6280992	20
RPFS	Reversions (Continuous)	2.270086	0.965382	5.338085	0.06	0.9256198	4
RPFS	Reversions (Continuous)	1.665084	0.4563628	6.075223	0.44	1.8512397	8
RPFS	Reversions (Continuous)	2.078035	0.634895	6.801485	0.23	2.7768595	12
RPFS	Reversions (Continuous)	1.522555	1.0765157	2.153404	0.017	3.7024793	16
RPFS	Reversions (Continuous)	1.104914	0.999559	1.221373	0.051	4.6280992	20

Table S3: Clinical history of 6 patients with *BRCA2* homozygous deletion and both-baseline and end-of-treatment samples, related to Figure 3.
EOT=end-of-treatment, PSA=prostate specific antigen, PCWG2=Prostate Cancer Working Group 2.

MANUSCRIPT PATIENT ID	Time on PARPi (days)	Prior treatments	Description of response (PSA)	Description of response (Radiology)	Description of progression	Comments
p21	1115	Docetaxel, Enzalutamide, Cabazitaxel	Deep PSA response, 539 ug/L at baseline with nadir of 2.2 ug/L	Measurable disease, best response partial response	Growth of a solitary liver lesion and retroperitoneal nodes. Otherwise treated liver and bone lesions.	EOT sample taken 48 days after stopping PARPi
p27	617	Abiraterone, Enzalutamide, Cabazitaxel, Enzalutamide/AZD5363	PSA response, 510 ug/L at baseline with nadir of 64 ug/L	Measurable disease, best response partial response	Partial response by RECIST, PD on bone scan with spinal cord compression shortly after	EOT sample taken 21 days after stopping PARPi
p40	224	Docetaxel, Radium-223 and Enzalutamide	PSA response, 2980 ug/L at baseline with nadir of 574 ug/L	Evaluable disease, response in bone lesions on WBMRI, SD by PCWG2	Mixed bone response, new lung and pleural lesions	EOT sample taken 29 days after stopping PARPi
p23	503	Docetaxel, Enzalutamide, Cabazitaxel	PSA response, 456 ug/L at baseline with nadir of 30 ug/L	Measurable disease, best response partial response	Progression of bone, nodal and local disease	EOT sample taken 28 days after stopping PARPi
p31	320	Docetaxel, Abiraterone, Radium-223, Cabazitaxel	PSA response, 461 ug/L at baseline with nadir of 94 ug/L	Evaluable disease, mixed response in bone lesions, SD by PCWG2	Ongoing mixed response, with one lesion causing spinal cord compression	EOT sample taken 27 days after stopping PARPi
p32	448	Docetaxel, Enzalutamide	PSA response, 89 ug/L at baseline with nadir of 26 ug/L	Measurable disease, best response partial response	New nodal disease and increasing bone disease	EOT sample taken on the day of stopping PARPi
p29	505	Abiraterone, Docetaxel, Enzalutamide/BI 836845	PSA response, 14 ug/L at baseline with nadir of 1.8 ug/L	Measurable disease, best response partial response	Progression of nodal disease	EOT sample taken 22 days after stopping PARPi
p25	644	Abiraterone, Docetaxel	PSA response, 107 ug/L at baseline with nadir of 6.1 ug/L	Measurable disease, best response partial response	Oligoprogression at L5 requiring radiotherapy. Maintained response elsewhere	EOT sample taken 28 days after stopping PARPi
p39	No in-depth clinical data available					

Table S4: Evaluable samples for *BRCA2* homozygous deletion validation experiments, related to Figure 3. Data split by assay type. BL=baseline, EOT=end-of-treatment, FFPE=formalin-fixed paraffin embedded.

	ctDNA WGS Evaluable		Tissue Sample Detail (Date, Type)		Tissue FISH Evaluable		Tissue RNAish Evaluable	
Patient ID	BL	EOT	Pre-Treatment	EOT	Pre-Treatment	EOT	Pre-Treatment	EOT
p21	Yes	Yes	19/11/2015, Fresh Frozen Biopsy	Not Available	Yes	Not Available	Yes	Not Available
p27	Yes	Yes	04/04/2016, Fresh Frozen Biopsy	Not Available	Yes	Not Available	Yes	Not Available
p40	Yes	Yes	21/09/2012, Diagnostic FFPE Biopsy	Not Available	Yes	Not Available	Yes	Not Available
p23	Yes	Yes	20/07/2016, Fresh Frozen Bopsy	28/03/2018, Fresh Frozen Biopsy	Yes	Yes	Yes	Yes
p29	Low Purity	Yes	30/09/2009, Diagnostic FFPE Biopsy	Not Available	QC Fail	Not Available	QC Fail	Not Available
p25	Yes	Low Purity	24/05/2017, Fresh Frozen Biopsy	Not Available	Yes	Not Available	Yes	Not Available
p31	Yes	Yes	02/10/2017, Fresh Frozen Biopsy	Not Available	Yes	Not Available	QC Fail	Not Available
p32	Yes	Yes	23/11/2017, Fresh Frozen Biopsy	Not Available	QC Fail	Not Available	QC Fail	Not Available
p39	Low Purity	Yes	07/03/2017, Diagnostic FFPE Biopsy	Not Available	Yes	Not Available	Yes	Not Available
